# A review of omics studies in sarcopenia: from molecular mechanisms to hepatic-gut-muscle interactions in chronic liver disease comorbidity

**DOI:** 10.3389/fcimb.2025.1710582

**Published:** 2026-01-06

**Authors:** Xiaohui Xue, Jun Xu, Huijuan Wang, Kainan Wang, Yumu Chen, Yiting Xu, Shuping Que, Zhengtao Liu

**Affiliations:** 1 Key Laboratory of Artificial Organs and Computational Medicine in Zhejiang Province, Shulan International Medical College, Zhejiang Shuren University, Hangzhou, China; 2 School of Medicine, Zhejiang Chinese Medical University, Hangzhou, China; 3 NHC Key Laboratory of Combined Multi-Organ Transplantation, Key Laboratory of the Diagnosis and Treatment of Organ Transplantation, First Affiliated Hospital, School of Medicine, Zhejiang University, Hangzhou, China; 4 Key Laboratory of Organ Transplantation, First Affiliated Hospital, School of Medicine, Zhejiang University, Hangzhou, China; 5 Division of Hepatobiliary and Pancreatic Surgery, Department of Surgery, First Affiliated Hospital, School of Medicine, Zhejiang University, Hangzhou, China; 6 Department of Hepatobiliary Surgery, Shulan (Hangzhou) Hospital, Shulan International Medical College, Zhejiang Shuren University, Hangzhou, China; 7 Ya-er-zhuang Clinics, Hangzhou, China

**Keywords:** aging, chronic liver disease, gastrointestinal flora imbalance, gut-muscle axis, muscle-liver axis, omics analysis, sarcopenia

## Abstract

Sarcopenia is an aging-related skeletal-muscle disorder characterized by progressive loss of muscle mass, strength, and function, and it frequently co-occurs with chronic liver disease (CLD) and other comorbidities. Conventional approaches struggle to resolve its pronounced heterogeneity, whereas multi-omics technologies now offer a systematic, molecular-level avenue to dissect its pathogenesis. By integrating ten omics studies of sarcopenia and six of CLD-associated sarcopenia, we propose a dual-layer “commonality–specificity” framework. At the level of commonality, we identify four core pathological pillars: proteostasis imbalance, mitochondrial dysfunction, chronic inflammation, and dysregulation of the gut–muscle axis. At the specificity level, focusing on the CLD context, we observe that these networks are selectively perturbed within the liver-disease microenvironment, leading us to advance the “cooperative accumulation of multiple weak signals” hypothesis to explain how multi-axis crosstalk drives muscle wasting in this setting. To date, omics findings remain largely correlational, posing challenges for clinical translation. Future investigations should integrate cutting-edge technologies—such as single-cell multi-omics, spatial transcriptomics, and computational modeling—to shift the research paradigm from static profiling to dynamic mechanistic dissection and precision intervention. This review provides both a theoretical foundation and a developmental roadmap for comprehensively understanding the mechanisms underlying sarcopenia comorbidities and for achieving precision diagnosis and treatment.

## 1 Introduction

Sarcopenia is a systemic skeletal-muscle disorder characterized by progressive loss of muscle mass, strength, and function, which markedly elevates the risks of falls, fractures, cognitive impairment, and mortality. Skeletal muscle mass remains stable until the age of 45 years and then declines substantially, with lower-limb muscles deteriorating more rapidly than upper-limb muscles (Janssen et al., 2000). Since Rosenberg coined the term in 1989 (Rosenberg, 1989), the definition of sarcopenia has undergone multiple iterations, with diagnostic criteria issued by the European Working Group on Sarcopenia in Older People (EWGSOP) (Cruz-Jentoft et al., 2010; Cruz-Jentoft et al., 2018), the Foundation for the National Institutes of Health (FNIH) (Studenski et al., 2014), the International Working Group on Sarcopenia (IWGS) (Fielding et al., 2011) and the Asian Working Group for Sarcopenia (AWGS) (Chen et al., 2014) (**Table 1**). Among these, the EWGSOP2 definition is the most widely adopted and has been incorporated into ICD-10-CM (M62.84). In this definition, low muscle strength is the primary criterion, while low muscle quantity/quality and poor physical performance are used for grading (Cruz-Jentoft et al., 2018).

**Table 1:** Diagnostic criteria for sarcopenia.

Diagnostic criteria for sarcopenia	Diagnostic core	Diagnostic methods	Muscle strength cutoff	Muscle mass cutoff	Physical function cutoff	Screening method	Classification criteria	Special notes
EWGSOP (2010)	(1)Muscle Mass (2)Muscle strength or Physical function	DXA, BIA; Handgrip dynamometer; Gait speed, Short Physical Performance Battery.	Grip strength: Men <30kg Women <20kg	(1)DXA: Men <7.26 kg/m^2^ Women <5.5 kg/m^2^ (2)BIA: Men <8.87 kg/m^2^ Women <6.42 kg/m^2^	Gait speed < 0.8 m/s SPPB ≤8 points	N/A	(1) Possible sarcopenia: Reduced muscle strength only. (2) Confirmed sarcopenia: Reduced muscle strength + reduced muscle mass. (3) Severe sarcopenia: Reduced muscle strength + reduced muscle mass + reduced physical function.	(1) Must consider comorbidities and individual variability that may explain findings. (2) Algorithm also applies to at-risk younger individuals.
EWGSOP2 (2018)	Muscle strength	DXA, BIA; Jamar dynamometer, 5-TCS; Gait speed, SPPB, TUG, 400-meter walk test.	(1)Grip strength: Men <27kg Women <16kg (2)5-TCS: ≥15 seconds	(1)ASM: Men <20kg Women <15 kg (2)ASM/height^2^:Men < 7.0 kg/m^2^ Women < 5.5 kg/m^2^	Gait speed <0.8m/s SPPB ≤8 points TUG >20 seconds 400-meter walk: Incomplete or ≥6 points	SARC-F questionnaire or clinical suspicion	(1) Confirmed sarcopenia: Reduced muscle strength + reduced muscle mass. (2) Severe sarcopenia: Reduced muscle strength + reduced muscle mass + reduced physical function.	ICD-10 code (M62.84)
AWGS	Muscle strength	DXA, BIA; hand-held dynamometer; 6-m usual gait speed.	Grip strength: Men <26kg Women <18kg	(1)BIA: Men < 7.0 kg/m^2^ Women < 5.7 kg/m^2^ (2)DXA: Men < 7.0 kg/m^2^ Women < 5.4kg/m^2^	Gait speed ≤0.8 m/s	SARC-F questionnaire	(1) Possible sarcopenia: Reduced muscle strength or reduced physical function. (2) Confirmed sarcopenia: Possible sarcopenia + reduced muscle mass (3) Severe sarcopenia: Confirmed sarcopenia + reduced physical function.	Adjusted thresholds for Asian populations
FNIH	Muscle strength	DXA, BIA; Hand-held dynamometer; Gait Speed.	Grip strength: Men <26kg Women <16kg	ALM/BMI: Men <0.789 Women <0.512	Gait speed < 0.8 m/s	N/A	Confirmed sarcopenia: Requires both low muscle mass and low muscle strength.	Emphasizes exclusion of obesity effects
IWGS	(1) Physical function (2) Muscle mass	DXA; 6-m usual gait speed.	N/A	DXA: Men <7.23 kg/m^2^ Women <5.67 kg/m^2^	Gait speed <1.0 m/s	N/A	Confirmed sarcopenia: Requires both reduced muscle mass and reduced physical function.	Emphasis on functional performance

EWGSOP, The European Working Group on Sarcopenia in Older People; AWGS, The Asian Working Group for Sarcopenia; FNIH, The Foundation for the National Institutes of Health; IWGS, The International Working Group on Sarcopenia; DXA, Dual-Energy X-ray Absorptiometry; BIA, Bioelectrical lmpedance; ASM, AppendicularSkeletal Muscle Mass; ALM, Appendicular LeanMass; BMl, Body Mass Index; SPPB, Short PhysicalPerformance Battery; 5-TCS, 5-time chair stand; TUG, Timed Up and Go Test; SARC-F questionnaire, Strength; Assistance with walking, Rise from a chair, Climb stairs, and Falls questionnaire; N/A, “Not Applicable” or “Not Available “.

Recently, the Global Leadership Initiative on Sarcopenia (GLIS) established a conceptual definition of sarcopenia through an international Delphi study, clarifying that sarcopenia is a systemic skeletal-muscle disorder whose conceptual framework must incorporate three core elements: muscle mass, muscle strength, and specific muscle force. Moreover, the consensus explicitly categorizes physical performance as a consequence rather than a component of sarcopenia. The definition further emphasizes that the prevalence of sarcopenia increases with age and that the condition is potentially reversible. Building on this conceptual framework, GLIS plans to develop operational diagnostic criteria applicable to both clinical practice and research settings (Kirk et al., 2024).

Importantly, sarcopenia is not restricted to older adults; prolonged immobilization, malnutrition, or comorbidities such as diabetes, cancer, and chronic heart failure can also induce secondary sarcopenia (Kizilarslanoglu et al., 2016).

Owing to variations in diagnostic criteria and population characteristics, the reported prevalence of sarcopenia exhibits substantial heterogeneity. In 2018, the global prevalence was 5–13% among adults aged 60–70 years and reached ~50% among those aged> 80 years (Morley, 2012). It is projected that by 2050, sarcopenia will affect the health of 500 million older individuals worldwide (Cruz-Jentoft et al., 2010). Among hospitalized older adults, direct and indirect healthcare expenditures for individuals with sarcopenia were roughly fivefold higher than for those without the condition (Antunes et al., 2017). Collectively, sarcopenia imposes a severe strain on healthcare resources and represents a substantial socioeconomic burden.

Computed tomography (CT) and magnetic resonance imaging (MRI) are widely recognized as the gold standards for assessing muscle mass. The primary measured parameters include the skeletal muscle area (SMA) at the third lumbar vertebra, the height-normalized skeletal muscle index (SMI), and the muscle radiation attenuation (MRA). Dual-energy X-ray absorptiometry (DXA) has been clinically validated as an alternative modality. Bioelectrical impedance analysis (BIA) and musculoskeletal ultrasonography are suitable for clinical screening, although their measurement accuracy is limited. Muscle function is primarily evaluated using the following internationally accepted indicators: 1) handgrip strength test; 2) 5-time chair stand (5-TCS); 3) usual gait speed assessment; 4) Short Physical Performance Battery (SPPB); and 5) the Strength, Assistance with walking, Rise from a chair, Climb stairs, and Falls (SARC-F) questionnaire (Cruz-Jentoft et al., 2018). The specific diagnostic criteria are presented in **Table 1**.

Currently, exercise and nutrition remain the cornerstone of sarcopenia management, and no precision pharmacological targets have yet been established.

Chronic liver disease (CLD), characterized by hepatocellular injury and impaired hepatic function, has emerged as one of the leading global causes of mortality and disability. Since 2000, its overall mortality has continued to rise, accounting for approximately two million deaths annually and imposing a substantial burden on global public-health systems (Asrani et al., 2019). The Global Burden of Disease (GBD) 2019 study stratifies chronic liver diseases into subtypes, including viral hepatitis (HBV/HCV), alcohol-associated liver disease (ALD), metabolic dysfunction-associated steatotic liver disease (MASLD) (Xiao et al., 2025), liver cancer, and other chronic liver conditions, with underlying data publicly accessible via the GHDx platform (https://ghdx.healthdata.org/).

Clinical evidence has unequivocally established a robust bidirectional association between CLD and sarcopenia. A recent single-center study enrolling 151 patients with CLD found that 60% were pre-sarcopenic, 30% were already sarcopenic, and 50% exhibited osteopenia; sarcopenia was significantly associated with elevated liver frailty index (LFI) scores, underscoring a synergistic link between musculoskeletal deficits and hepatic vulnerability (Reichelt et al., 2024). Pooled analyses indicate that the overall prevalence of sarcopenia among cirrhotic patients is 33%, rising further to 38% in those with concomitant malignancy. Notably, this association displays a pronounced sex disparity, with male patients exhibiting a markedly higher risk than females (Mazeaud et al., 2023).

Accumulating clinical evidence demonstrates that sarcopenia significantly influences disease progression and clinical outcomes in patients with chronic liver disease. Systematic analyses indicate that sarcopenia not only increases the risk of developing MASLD and its accompanying hepatic fibrosis but also raises mortality and readmission rates in patients with cirrhosis (Thyloor Kenchappa et al., 2023; Malik et al., 2024). A recent meta-analysis further establishes sarcopenia as an independent predictor of 28-day mortality in patients with acute-on-chronic liver failure (ACLF) (He et al., 2025). Among individuals with HCC, sarcopenia has likewise been identified as an independent correlate of adverse prognosis across diverse therapeutic modalities (Cespiati et al., 2024).

Traditional approaches, constrained by the examination of single pathways or a limited set of biomarkers, are insufficient to capture the marked heterogeneity of sarcopenia or its multi-systemic interactions with chronic liver disease. Recent advances in omics technologies—spanning genomics, epigenomics, transcriptomics, proteomics, metabolomics, and microbiomics—now offer an unprecedented, systems-level perspective for elucidating the molecular mechanisms underlying disease. Multi-omics integration, in particular, has emerged as a pivotal strategy to overcome methodological bottlenecks by systematically combining molecular data across multiple biological layers. Of the 10 omics-based sarcopenia studies reviewed herein, 6 employed integrative multi-omics approaches, substantially enhancing the resolution of molecular network interactions. Notably, metabolomics—by virtue of capturing real-time biochemical activity and physiological states—is considered the omics layer closest to the “molecular phenotype,” enabling precise detection of sarcopenia-associated metabolic perturbations and their dynamic correlation with disease severity (Marques et al., 2023).

This review synthesizes 10 omics studies on sarcopenia and 6 on sarcopenia secondary to CLD, systematically analyzing multidimensional data to elucidate its pathogenesis and to provide a comprehensive theoretical framework for precision diagnosis and therapy. The manuscript is organized in three sequential sections: construction of a multi-system molecular map of sarcopenia; in-depth dissection of comorbidity-specific mechanisms in the context of CLD; and critical appraisal of omics-guided therapeutic strategies and remaining challenges. Contemporary research has moved beyond single-tissue pathology, increasingly emphasizing the central role of multi-organ crosstalk. Against this backdrop, the present review highlights the interplay between the muscle–liver and gut–muscle axes in CLD-associated sarcopenia.

Unlike previous sarcopenia reviews that merely catalogued disparate omics findings, the present work adopts a markedly different analytical perspective. We propose a dual-layer “commonality–specificity” interpretative framework: at the commonality level, we integrate potential interaction networks among proteostasis, mitochondrial function, and inflammation across multiple systems; at the specificity level, we focus on the particular pathological context of CLD to dissect how these networks evolve selectively under comorbid conditions. Based on a systematic appraisal of current evidence, we further posit that the pathogenesis of CLD-related sarcopenia may follow a “cooperative accumulation of multiple weak signals” model driven by the liver-disease microenvironment. This framework offers an integrative conceptual lens for comprehending the pathophysiology of such complex comorbidities.

## 2 A multi-system molecular framework for sarcopenia: an integrative omics perspective

The pathogenesis of sarcopenia is highly intricate, encompassing perturbed protein turnover, mitochondrial dysfunction, chronic low-grade inflammation, and degeneration of the neuromuscular unit (Wozniak and Anderson, 2009; Gielen et al., 2015; Marzetti et al., 2017; Cruz-Jentoft et al., 2018). Multi-omics technologies now provide a panoramic vantage point for systematically dissecting this multifaceted network. By integrating ten independent studies (**Table 2**; details are summarized in **Supplementary Table S1**), this section aims to construct a core pathological framework that spans molecular, cellular, and organ-level hierarchies. A collective reading of these studies consistently highlights proteostatic imbalance and mitochondrial dysfunction as pivotal events in sarcopenia. Omics datasets further dissect the contribution of systemic inflammaging, the long-range regulatory role of the gut–muscle axis, and the impact of circulating mediators such as bile acids and endocrine hormones. The discussion below follows this organizational logic. The trans-omics evidence pertaining to the core pathological mechanisms of sarcopenia has been synthesized and consolidated in **Supplementary Table S2**.

**Table 2:** Overview of core omics studies informing the multi-system molecular framework of sarcopenia.

Author	Year	Sample size	Types of omics analysis	Main findings
Zeng Zhang	2025	80	Metagenomic Analysis	1.Niacin produced by *Bifidobacterium adolescentis* elevates NAD^+^ levels, activates the SIRT1/PGC-1α axis, and promotes mitochondrial biogenesis, thereby increasing oxidative myofibers in skeletal muscle, preventing muscle atrophy, and improving muscle health in sarcopenic patients.
			Metabolomics Analysis	2.*Bifidobacterium* and *Collinsella* were specifically enriched in healthy individuals and sarcopenic individuals, respectively. In sarcopenia patients, beneficial species such as *Bifidobacterium longum, Bifidobacterium pseudocatenulatum*, and *Bifidobacterium adolescentis* were markedly reduced.
Lin Yin	2025	≤25*	Proteomic Analysis	The key genes associated with sarcopenia include *Apoal, Apoe, Apoa 2, Rpl 21, Nefm, Cd 9, Rpl 6*, and *Cd 81*. Among them, CD 9 may be a new biomarker for sarcopenia. Dapoxetine, levomilnacipran, and milnacipran can target CD9 and are expected to become drugs for the treatment of sarcopenia.
			Transcriptomics Analysis	
Yan Guo	2024	15	Metabolomics Analysis	1.The intestinal microbial community of individuals with low grip strength is more diverse, but the α-diversity and β-diversity are lower. The relative abundances of Parabacteroides and Intestinibacter in the low grip strength group have significantly increased.
			Microbiome Analysis	2.The levels of serum cinnamic acid and its derivatives in the low grip strength group were significantly lower. There were significant differences in the biosynthetic pathways of phenylalanine, tyrosine and tryptophan between the low grip strength group and the normal grip strength group.
Melissa R. Pergande	2024	12	Proteomic Analysis	1.With increasing age, significant changes occur in the proteins and metabolites related to energy metabolism, fatty acid β-oxidation, and muscle structure in skeletal muscle.
			Metabolomics Analysis	2.It is mainly manifested that energy metabolism pathways and pathways related to muscle structure and contraction are significantly down -regulated in elderly animals, and lipid and carbohydrate metabolism pathways are also altered. These changes lead to a decline in muscle strength by affecting muscle energy supply and altering muscle structure and function.
Xinrong Zuo	2023	60	Transcriptome Analysis	1.In sarcopenia, pathways such as glycolysis, the tricarboxylic acid cycle, fatty-acid metabolism, and branched-chain amino-acid catabolism are impaired.
			Proteomics Analysis	2.In sarcopenia patients, the *BCAT2* and *BCKDHB* genes involved in BCAA metabolism are significantly downregulated, and the levels of the key enzymes BCAT2 and BCKDHB are markedly reduced.
			Metabolomics Analysis	3.Dysfunction in BCAA catabolism may be a driver of sarcopenia and is positively correlated with muscle mass and grip strength. Within this pathway, the BCKDHB and BCAT2 genes serve as potential therapeutic targets; activating BCKDH with BT2 to enhance BCAA catabolism can improve muscle mass and function, while inhibiting mTOR activation counteracts BCAA-associated skeletal muscle atrophy.
Jair Marques	2023	22	Metabolomic Analysis	1.After FDR correction, Cit was the sole plasma metabolite exhibiting a significant difference between the two groups, with levels significantly higher in sarcopenic than in non-sarcopenic individuals.
Li Guan	2023	15	Transcriptomic Analysis	1.The level of butyrate in fecal samples from the sarcopenia group was significantly reduced.
			Metabolomics Analysis	2.Butyrate may promote the proliferation of C2C12 myoblasts by activating the ERK/MAPK signaling pathway and upregulating the expression of Myf5 and MyoD, thereby enhancing the regenerative capacity of skeletal muscle to a certain extent.
			Microbiome Analysis	
Yangli He	2023	63	Shotgun Metagenomic Sequencing	1.In sarcopenia patients, beneficial taxa—including Bifidobacterium pseudocatenulatum, Bifidobacterium longum, Bifidobacterium adolescentis, Phascolarctobacterium faecium, and Faecalibacterium prausnitzii—were markedly reduced, whereas the relative abundance of Phascolarctobacterium succinatutens was increased.
			Metabolomics Analysis	2.Phascolarctobacterium faecium can promote the production of shikimate, thereby influencing the biosynthesis of phenylalanine, tyrosine, and tryptophan and subsequently impacting clinical indicators.
Rafael Opazo	2021	41	Metabolomics Analysis	Plasma metabolites differ markedly between sarcopenic and non-sarcopenic older adults, and these differences chiefly affect amino-acid- and lipid-related pathways, including amino-acid biosynthesis, arginine and proline metabolism, biosynthesis of ornithine-derived alkaloids, linoleic-acid metabolism, and the synthesis of unsaturated fatty acids.
Yanxia Lu	2020	189	Transcriptomics Analysis	1.In sarcopenia patients, the proto-oncogene serine/threonine-protein kinase PIM1 is the most markedly down-regulated gene. PIM1 kinase interacts with the DNA-binding domain of the vitamin D receptor and participates in the 25(OH)D_3_ signaling pathway.
			Metabonomics Analysis	2.The malnutrition defined by a low MNA score was independently associated with sarcopenia, and the nutritional status was mainly related to the maintenance of muscle mass, and to some extent, also related to muscle strength.

*The specific quantity varies depending on the experiment; BCAA, Branched-chain amino acid; FDR, False discovery rate; Cit, Citrulline; IGF-I, Insulin-like Growth Factor-I; VLC-FA, Very-Long-Chain Fatty Acid; MNA, Mini Nutritional Assessment; BT2, a BCKDH inhibitor.

### 2.1 Proteostatic imbalance

Skeletal muscle protein turnover—the core physiological process sustaining muscle homeostasis—is governed by the dynamic equilibrium between muscle protein synthesis (MPS) and muscle protein breakdown (MPB) (Breen and Phillips, 2011). At the molecular level, MPB is primarily executed through two key pathways: proteasome–ubiquitin system-mediated proteolysis and lysosome-mediated autophagy (Fan et al., 2016). Notably, under atrophic conditions, the expression levels of transcription factors and effector proteins associated with both degradative pathways are markedly up-regulated (Bodine et al., 2001; Gomes et al., 2001; Cai et al., 2004).

At the transcriptomic level, Lu and colleagues employed genome-wide expression profiling to systematically analyze peripheral blood samples from 189 community-dwelling older adults (Lu et al., 2020). Rigorous differential expression analysis identified 196 statistically significant differentially expressed genes (DEGs). Most strikingly, nine of the ten most down-regulated genes (90%) were directly implicated in the metabolic regulation of threonine and/or lysine. Subsequent pathway enrichment analyses revealed significant over-representation of protein-metabolism-related pathways, including the mammalian target of rapamycin (mTOR) signaling cascade, the ubiquitin–proteasome degradation system, and the Protein Kinase A (PKA) signaling network. Importantly, the mTOR pathway—an atypical threonine/lysine kinase signaling axis—was markedly suppressed in individuals with sarcopenia (Lu et al., 2020).

Metabolomic findings corroborate the transcriptomic observations. Among the nine essential amino acids (EAAs), seven—including methionine, lysine, phenylalanine, threonine, and the branched-chain amino acids leucine, isoleucine, and valine—exhibited significantly reduced plasma concentrations (Lu et al., 2020). This finding carries substantial pathophysiological relevance, particularly because leucine, a critical activator of mTOR complex 1 (mTORC1), may directly suppress mTOR signaling when its availability is diminished (Bajotto et al., 2011). Given the central role of the mTOR pathway in modulating protein synthesis and preserving muscle protein homeostasis (Ilha et al., 2018), these multi-omics insights elucidate, at the molecular level, the mechanisms underlying disrupted muscle protein metabolism in sarcopenia and provide a robust theoretical foundation for the development of targeted nutritional interventions.

Furthermore, during the pathogenesis and progression of sarcopenia, dysregulated amino acid metabolism may impair muscle protein synthesis and degradation. Metabolomic studies have revealed distinct patterns of amino acid metabolic disturbances in affected individuals. Using untargeted metabolomics, Opazo and colleagues compared the plasma metabolomic profiles of sarcopenic (n = 20) and age-matched healthy control participants (n = 21) aged 60 years or older, revealing significant inter-group differences in six of the 20 proteinogenic amino acids. Specifically, sarcopenic individuals exhibited markedly elevated glutamine and methionine concentrations, whereas levels of leucine, glutamate, and other proteinogenic and non-proteinogenic amino acids were significantly reduced (Opazo et al., 2021).

This metabolic signature can be explained by two interrelated biological mechanisms. First, glutamine—the most abundant free amino acid in skeletal muscle, accounting for approximately 60% of the intracellular free amino-acid pool—is normally maintained at a concentration markedly higher than that in plasma. When muscle proteolysis is accentuated, large quantities of stored glutamine are released into the circulation, leading to a pronounced rise in plasma levels. Second, ammonium ions generated during amino-acid catabolism are taken up by peripheral tissues and, under the catalysis of glutamine synthetase, converted to glutamine that is subsequently released into the bloodstream. This process further elevates plasma glutamine while depleting the glutamate substrate, thereby lowering circulating glutamate concentrations. Collectively, these observations indicate that the protein-metabolism derangement in sarcopenia is characterized by a net imbalance of protein turnover driven predominantly by enhanced MPB (Opazo et al., 2021).

Previous studies have established FoxO3 as the master transcriptional regulator of skeletal muscle proteolysis. By directly binding to the promoters of autophagy-related genes such as LC3b, Gabarapl1, and Atg12l and by inducing the expression of E3 ubiquitin ligases Atrogin-1 and MuRF-1, FoxO3 coordinately activates both the lysosomal–autophagy pathway—accounting for approximately 70% of the increment in protein degradation—and the ubiquitin–proteasome system, thereby accelerating myofibrillar protein breakdown and driving muscle atrophy (Zhao et al., 2007; Milan et al., 2015). Animal experiments further validated this mechanism: compared with young controls, the expression of FoxO3, Atrogin-1, and MuRF-1 was significantly elevated in the tibialis anterior (TA), soleus (SOL), and extensor digitorum longus (EDL) muscles of aged mice (Zhang et al., 2025). Consequently, the hyperactivation of FoxO3-mediated proteolytic pathways constitutes the key molecular basis for the “enhanced breakdown” component of the disrupted protein turnover characteristic of sarcopenia.

Owing to 93% genomic homology between rhesus macaques and humans (Francis et al., 2008; Yan et al., 2011) and the pronounced susceptibility of the vastus lateralis to age-related type II fiber loss (Colman et al., 2005), Pergande et al. applied mass-spectrometry-based multi-omics to this model and documented marked reductions in actin, myosin, integrin, and collagen within the aged vastus lateralis—most pronounced between middle and old age (Pergande et al., 2024). These findings provide direct evidence of structural-protein depletion at the primate level and further substantiate the role of structural-protein loss as a central driver of sarcopenia.

In summary, disruption of skeletal-muscle proteostasis is widely recognized as a central pathogenic hallmark of sarcopenia, and this process is inextricably linked to mitochondrial failure. As the hub of cellular energy metabolism, mitochondrial decline precipitates an energetic crisis that subsequently perturbs protein turnover. The following section, therefore, focuses on the pivotal roles of mitochondrial dysfunction and impaired organellar quality control in the development of muscle wasting.

### 2.2 Energy crisis: mitochondrial dysfunction and quality-control deregulation

Skeletal-muscle function critically relies on the mitochondrial energy metabolism and quality-control systems. In the initiation and progression of sarcopenia, mitochondrial dysfunction operates as a central pathogenic mechanism, manifesting primarily as two intertwined anomalies: deranged energy metabolism and impaired quality control. These aberrations interact through an intricate molecular network to orchestrate the progressive deterioration of skeletal-muscle structure and function. Hereafter, leveraging multi-omics approaches, we systematically delineate the multi-omics evidence underlying mitochondrial dysfunction in sarcopenia and elucidate its pathological significance with respect to the aforementioned core processes.

#### 2.2.1 Impaired branched-chain amino acid (BCAA)-catabolizing enzymes mediate BCAA accumulation and chronically activate mTOR-driven energy-metabolic derangement

Zuo et al. performed multi-omics profiling of vastus lateralis biopsies obtained from 20 sarcopenic, 20 possibly sarcopenic, and 20 healthy aged individuals. Integrated transcriptomic and metabolomic analyses revealed pronounced alterations in branched-chain amino acid (BCAA) catabolism, the TCA cycle, fatty-acid degradation, glycolysis, and pyruvate metabolism during sarcopenia progression. Specifically, the expression of the rate-limiting BCAA catabolizing enzymes BCAT2 and BCKDHB was markedly reduced in sarcopenic muscle, leading to intramuscular accumulation of BCAAs and branched-chain α-keto acids (BCKAs). The accumulation of BCAAs chronically activated mTOR, leading to global downregulation of mitochondrial respiratory-chain genes and a significant decline in ATP, ADP, and phosphocreatine concentrations. Pharmacological intervention with rapamycin or BT2 (a BCKDH activator) concurrently suppressed mTOR signaling and restored mitochondrial function. Moreover, sustained mTOR activation further compromised insulin sensitivity and autophagic flux, as detailed in Section 2.2.5 (Zuo et al., 2025). Collectively, down-regulation of BCAA-catabolizing enzymes in sarcopenic skeletal muscle promotes the accumulation of BCAAs and BCKAs, chronically activates the mTOR pathway, suppresses respiratory-chain gene expression, and precipitates a drastic fall in ATP production and mitochondrial structural integrity, thereby driving progressive skeletal-muscle deterioration centered on mitochondrial dysfunction.

#### 2.2.2 Mitochondrial fatty-acid oxidation dysfunction disrupts energy homeostasis

Mitochondria constitute the principal site for the β-oxidation of long-chain fatty acids (LC-FA). Untargeted plasma metabolomics in a hip-fracture sarcopenia cohort (n = 22) revealed marked elevations in very-long-chain fatty acids (VLC-FAs) and their ω-oxidation products, dicarboxylated acyl-carnitines (Carn.DC). Orthogonal and sparse partial least squares discriminant analyses (oPLS-DA and sPLS-DA) identified NEFA 26:2 and Carn. 10.DC is the top discriminatory metabolite between sarcopenic and non-sarcopenic individuals. Metabolite-set enrichment analysis further indicated significant enrichment of mitochondrial fatty-acid oxidation impairment pathways, including carnitine palmitoyltransferase II deficiency and LCHAD deficiency (Marques et al., 2023). In addition, multi-omics profiling of skeletal muscle by Zuo and colleagues consistently demonstrated down-regulation of both downstream metabolites and key enzymes within the fatty-acid β-oxidation pathway (Zuo et al., 2025). Comparative plasma metabolomics performed by Opazo et al. showed significantly elevated baseline levels of ω-6 long-chain fatty acids—including linoleic acid (18:2n-6), γ-linolenic acid (18:3n-6), and arachidonic acid (20:4n-6)—in sarcopenic versus non-sarcopenic controls (Opazo et al., 2021). Collectively, these findings suggest a compensatory up-regulation of peroxisomal ω-oxidation alongside impaired mitochondrial β-oxidation in sarcopenia; although highly correlated, their causal interrelationship remains to be elucidated.

Multi-omics profiling of skeletal muscle from aged rhesus macaques further corroborates this notion (Pergande et al., 2024). In the vastus lateralis, palmitoylcarnitine and arachidonoylcarnitine accumulated markedly, whereas the β-oxidation end-product acetylcarnitine exhibited altered levels. Proteomic analyses revealed significant alterations in the expression of multiple enzymes integral to fatty-acid β-oxidation: enoyl-CoA isomerase 1 (ECI1) and the acetyl-CoA carboxylase carboxyl-transferase α-subunit 2 (THIM) were up-regulated, whereas ethylmalonyl-CoA decarboxylase (ECHD1) was down-regulated. Concurrently, proteomic data demonstrated a compensatory increase in enzymes involved in Adenosine Triphosphate (ATP) production—such as components of the electron-transport chain—along with downregulation of Adenosine Monophosphate (AMP)-metabolizing enzymes, underscoring an energy crisis in primate skeletal muscle. Integration of proteomic and metabolomic datasets disclosed an age-dependent, concerted dysregulation of the fatty-acid β-oxidation pathway. Furthermore, energy-metabolism–associated metabolites—including carnitine, trimethyllysine, phosphocreatine, and phosphatidylinositol 38:4 (PI 38:4)—were markedly decreased in middle-aged and older animals, corroborating the progressive decline in ATP-generating capacity and the resultant inability of aged rhesus macaques to sustain muscular strength and contractile function.

Taken together, current evidence indicates that mitochondrial fatty-acid oxidation dysfunction constitutes a conserved, cross-species mechanism underlying sarcopenia. Nevertheless, this hypothesis warrants further validation through more comprehensive molecular investigations and larger-scale clinical studies.

#### 2.2.3 ROS–ONOO^-^ axis-induced mtDNA damage underlies mitochondrial impairment

The mitochondrial electron transport chain (ETC) inevitably leaks superoxide anion (O_2_•^-^) and other reactive oxygen species (ROS) during oxidative phosphorylation. Ageing is accompanied by a progressive decline in antioxidant defenses, diminished ROS-scavenging capacity, and persistent ROS accumulation. This oxidative stress induces mtDNA mutations, which in turn exert feedback inhibition on ETC complex activity (Teixeira and Guariento, 2010). More critically, accumulated ROS compromise mitochondrial membrane integrity, triggering aberrant opening of the mitochondrial permeability transition pore (mPTP) and massive ROS efflux, thereby establishing a vicious cycle (Alway et al., 2017).

Notably, plasma metabolomic profiling in sarcopenic patients revealed a significant elevation in citrulline (Cit) levels. As citrulline is a by-product generated when inducible nitric oxide synthase (iNOS) converts L-arginine to nitric oxide (NO), its accumulation indicates heightened iNOS activity and a surge in NO production (Marques et al., 2023). Excess NO rapidly reacts with O_2_•^-^ leaked from the ETC, yielding the highly reactive oxidant peroxynitrite (ONOO^-^). ONOO^-^ irreversibly inhibits Complex I activity, severely compromising mitochondrial function and ultimately exacerbating cellular energy-metabolism crisis (Tengan et al., 2012).

#### 2.2.4 Decline in SIRT1/PGC-1α axis and dysregulation of CD9 jointly impair mitochondrial biogenesis

Silent information regulator 1 (SIRT1) orchestrates mitochondrial biogenesis by activating the peroxisome proliferator-activated receptor-γ coactivator-1α (PGC-1α), which in turn up-regulates nuclear respiratory factors 1 and 2 (NRF1/2) and subsequently induces the expression of mitochondrial transcription factor A (TFAM), ultimately promoting mtDNA replication and mitochondrial biogenesis (Anderson et al., 2008; Andres et al., 2015; Zhang et al., 2025). Consequently, the SIRT1–PGC-1α–NRF1/2–TFAM axis is recognized as the “master switch” governing mitochondrial biogenesis. In the mouse model, compared with young mice, the levels of SIRT1, PGC-1α, NRF1, NRF2, and TFAM in the anterior TA and SOL of aged mice, as well as the mtDNA/nDNA ratio, were significantly decreased, confirming the age-related inhibition of this axis (Zhang et al., 2025).

Genetic evidence further corroborates these findings: mice with muscle-specific deletion of PGC-1α exhibit sparse mitochondria, diminished exercise endurance, and premature-aging phenotypes. Conversely, muscle-specific overexpression of PGC-1α markedly protects against age-associated muscle atrophy (Handschin et al., 2007). In addition, NRF2 deficiency downregulates the expression of PGC-1α, NRF1, and TFAM and is positively correlated with reduced muscle strength and decreased mitochondrial content (Huang et al., 2019). Collectively, functional decline of the SIRT1–PGC-1α–NRF1/2–TFAM axis constitutes a central event underlying mitochondrial biogenic failure in sarcopenia and represents a promising therapeutic target. Future studies may orally co-administer SIRT1/PGC-1α agonists with NRF2 activators to rekindle the SIRT1–PGC-1α–NRF1/2–TFAM axis, followed by biomarker-stratified validation of their clinical efficacy in restoring mitochondrial function and muscle strength.

Notably, Lin and colleagues employed the SAM-P8 senescence-accelerated mouse model and, via integrated transcriptomic and proteomic profiling, identified CD9 as a pivotal gene strongly associated with sarcopenia. Immunofluorescence staining and quantitative PCR confirmed that CD9 expression in skeletal muscle declines significantly with advancing age. Gene set enrichment analysis (GSEA) further suggests that CD9 contributes to the onset and progression of sarcopenia by modulating mitochondrial biogenesis and oxidative phosphorylation (Yin et al., 2025). This novel finding opens a new avenue for elucidating the molecular mechanisms underlying sarcopenia. Future investigations should focus on delineating the precise molecular mechanisms by which CD9 governs mitochondrial function, and on assessing its evolutionary conservation in human sarcopenia as well as its potential as a novel biomarker or therapeutic target.

#### 2.2.5 Sustained mTOR activation suppresses autophagic flux and induces insulin resistance

As described in Section 2.2.1, accumulation of BCAAs in the sarcopenic skeletal muscle microenvironment chronically activates mTORC1. This hyper-activation accelerates mitochondrial damage and drives sarcopenia progression through two parallel and mutually reinforcing mechanisms: 1) Autophagic gate closure—hyper-phosphorylation of ULK1 at Ser757 disrupts the ULK1–AMPK complex, lowers the LC3-II/LC3-I ratio, promotes p62 accumulation, and blocks PINK1-Parkin-mediated mitophagic flux, thereby preventing the removal of damaged mitochondria; 2) Insulin signaling disruption—excessive phosphorylation of IRS1 at Ser307 attenuates IRS1–PI3K interaction, reduces AKT-Ser473 phosphorylation, impairs GLUT4 translocation, and decreases skeletal muscle glucose uptake. Transmission electron microscopy confirms a sharp decline in healthy mitochondria within muscle fibers, with the remaining organelles displaying swelling and disrupted cristae. Pharmacological interventions with rapamycin or BT2 concurrently relieve mTORC1-mediated dual suppression of autophagy and insulin signaling, restore mitophagic flux, enhance insulin sensitivity, and re-establish mitochondrial network integrity (Zuo et al., 2025). Collectively, BCAA-driven persistent activation of mTORC1 blocks autophagy initiation via ULK1-Ser757 phosphorylation and attenuates the insulin-Phosphatidylinositol 3-Kinase – Protein Kinase B (PI3K-AKT) axis through IRS1-Ser307 phosphorylation, resulting in the accumulation of damaged mitochondria, impaired glucose uptake, and reduced Adenosine Monophosphate-activated Protein Kinase (AMPK) -PGC-1α axis activity. These events precipitate mitochondrial quality decline and energy-metabolic derangement, establishing mTORC1 hyper-activation as the pivotal molecular hub driving progressive skeletal muscle deterioration in sarcopenia.

Collectively, multi-omics analyses underscore the fundamental role of mitochondrial dysfunction in sarcopenia. Targeting these mechanisms may disrupt the closed-loop mitochondrial energy crisis of sarcopenia, offering a novel paradigm for delaying—or even reversing—muscle aging.

Initial mitochondrial dysfunction and deranged quality-control systems create a self-reinforcing positive-feedback loop with ensuing low-grade inflammation that progressively amplifies tissue injury. Within this intricate interactome, chronic systemic low-grade inflammation operates as a pivotal pathogenic driver of sarcopenia. The following discussion, therefore, focuses on its mechanistic contribution to muscle wasting.

### 2.3 Systemic driver: chronic low-grade inflammation

Systemic, chronic low-grade inflammation (inflammaging) constitutes a fundamental pathological driver of sarcopenia initiation and progression. This persistent inflammatory milieu accelerates declines in muscle mass, strength, and function through multiple mechanisms. A large-scale genome-wide association study (GWAS) conducted within the UK Biobank (n = 181,301) identified specific human leukocyte antigen (HLA) genotypes that are significantly associated with sarcopenia risk, thereby providing robust genetic evidence for the pivotal role of immune–inflammatory responses in sarcopenia pathogenesis (Hernandez Cordero et al., 2019).

Multi-omics investigations have revealed that inflammation is a multi-layered, highly integrated hub in the initiation and progression of sarcopenia. At the transcriptomic level, pro-inflammatory genes (e.g., *TNFAIP3, CXCR4, NAMPT*) are significantly up-regulated in peripheral blood mononuclear cells, and the NF-κB signaling axis is distinctly activated, indicating an amplified systemic inflammatory response. Metabolomic profiling further demonstrates that plasma levels of several essential and branched-chain amino acids are reduced, presumably because immune cells competitively consume these nutrients under inflammatory conditions, thereby depriving muscle of anabolic substrates. Concurrently, functional vitamin B6 deficiency and decreased choline availability perturb one-carbon metabolism and the homeostasis of associated inflammatory metabolites, exacerbating the metabolically inflamed milieu. Moreover, elevated smoking-related nicotine metabolites have been shown to amplify oxidative stress and inflammatory reactions (Lu et al., 2020). These observations corroborate the transcriptomic findings of Zuo et al. in the vastus lateralis muscle, where MAPK, PI3K, NF-κB, and JAK-STAT inflammatory pathways were also broadly enriched (Zuo et al., 2025). Collectively, omics evidence indicates that chronic low-grade inflammation drives the progressive loss of muscle mass and function by simultaneously activating immune signaling, remodeling metabolic homeostasis, and potentiating protein catabolism.

Although emerging evidence has sketched an important role for inflammation in sarcopenia, the depth and systematic rigor of research in this area remain insufficient. Most studies to date were not specifically designed to elucidate inflammatory mechanisms; analyses remain largely descriptive and lack systematic integration of inflammatory signaling networks, followed by functional validation, which represents a critical bottleneck. Looking forward, multicenter, multi-omics integrative strategies are urgently needed to construct dynamic regulatory models of inflammatory signaling in sarcopenia. Such efforts will not only clarify core molecular mechanisms but also provide a solid theoretical foundation for developing pathway-specific, tissue-targeted precision anti-inflammatory interventions.

The core cellular pathogenesis of sarcopenia lies in proteostatic imbalance and mitochondrial dysfunction; however, chronic systemic low-grade inflammation acts not only as a direct insult to muscle but also as a systemic hub that integrates multiple pathological signals and drives these cellular aberrations. Within this network, the gut—an essential immuno-metabolic organ—serves as a critical upstream source: its dysfunction-induced barrier disruption and dysbiosis trigger and perpetuate systemic inflammation, thereby systemically fueling the onset and progression of sarcopenia. Consequently, the gut-mediated systemic pathomechanisms transmitted via the gut–muscle axis will be the focal point of the ensuing discussion.

### 2.4 Long-range regulation: the gut–muscle axis

In muscle health research, accumulating evidence indicates that structural disruption and functional impairment of the gut microbiota are closely linked to the onset and progression of sarcopenia. Building on this association, the “gut–muscle axis” concept has been formulated to systematically delineate the bidirectional communication between the intestinal microbiota and skeletal muscle, thereby offering a novel lens through which to understand the pathophysiology of sarcopenia. The trillions of microbial cells colonizing the gastrointestinal tract actively modulate a wide array of host physiological processes. By metabolizing dietary fibers, proteins, and other substrates into bioactive compounds, these microorganisms can directly or indirectly regulate muscle health (Guinane and Cotter, 2013). Dysbiosis can influence organ function at a distance by modulating microbial metabolites that intervene in key host signaling pathways. For example, metabolites such as butyrate and ursodeoxycholic acid have been shown to finely tune EGFR, VEGF, and PI3K/AKT/mTOR signaling—cascades central to both cancer biology and muscle metabolism—thereby modulating cellular energy flux, inflammatory responses, and tissue-repair processes (Gong et al., 2024).

**Figure 1:**
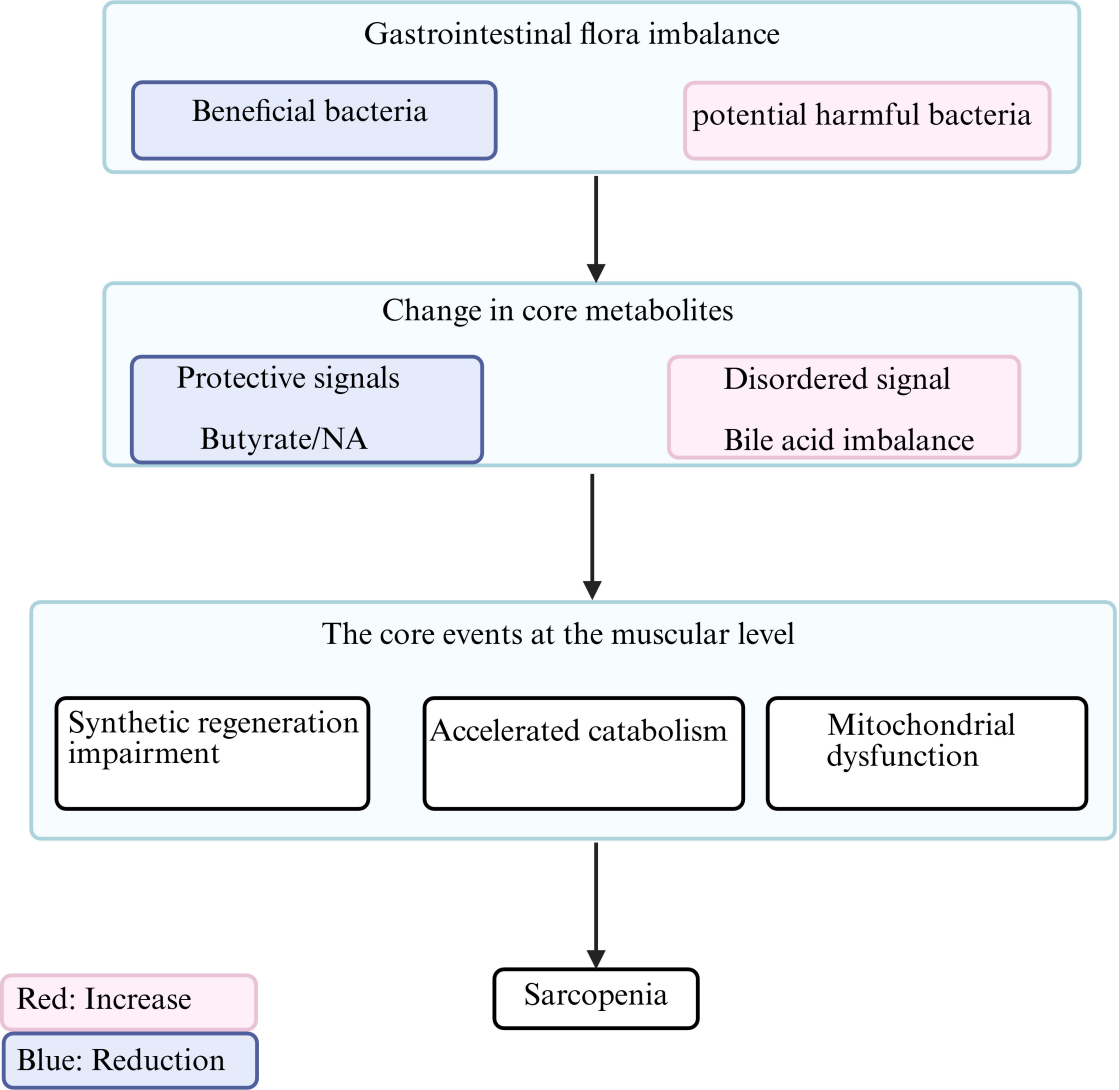
Schematic illustration of the core gut-muscle axis mechanisms in sarcopenia. NA, Nicotinic acid. (Created in BioRender. HUI, X. (2026) https://BioRender.com/z5qod0k).

This chapter will systematically dissect the characteristic alterations of the gut microbiota in individuals with sarcopenia, focus on the multi-dimensional interactions within the “microbiota–metabolite–muscle” axis (**Figure 1**), and—based on current evidence—evaluate the scientific rationale and translational prospects of targeted microbiome modulation as a potential therapeutic strategy.

#### 2.4.1 Signature patterns of gut microbiota dysbiosis in sarcopenia

Although several human studies report no significant differences in gut α-diversity (Shannon, Simpson, or Chao1 indices) between sarcopenic individuals and healthy controls (Guan et al., 2023; He et al., 2023; Zhang et al., 2025), accelerated-aging rats induced by D-galactose show the opposite pattern: richness (Chao1) and diversity (Shannon and Simpson) are markedly lower in the aged model group than in controls (He et al., 2023). Findings on β-diversity (community structure) are also inconsistent: some studies observe pronounced structural divergence between sarcopenic patients and healthy controls (He et al., 2023; Zhang et al., 2025), whereas others detect only modest separation with no significant sample clustering (He et al., 2023). Notably, older adults with low hand-grip strength (HGS) exhibit a distinctive microbial signature: despite increased species richness, α-diversity is reduced, indicating uneven community distribution, while diminished β-diversity suggests a more homogeneous and less ecologically heterogeneous microbiota (Guo et al., 2024). Collectively, sarcopenia-associated gut-microbiome alterations are characterized by shifts in community structure (β-diversity) and specific taxonomic abundance changes (e.g., reduced evenness in low-HGS individuals) rather than global α-diversity differences. Future work integrating metagenomics with functional assays—such as fecal microbiota transplantation or targeted metabolite profiling—is required to establish the causal roles and molecular mechanisms of key microbial taxa in sarcopenia.

Key microbial alterations include: 1) a pronounced depletion of beneficial taxa, including *Bifidobacterium*—particularly the short-chain-fatty-acid (SCFA)-producing strains *B. longum, B. pseudocatenulatum*, and *B. adolescentis* (Heinken et al., 2014; Turroni et al., 2016; He et al., 2023; Zhang et al., 2025). Among these, the abundance of *B. adolescentis* correlates positively with multiple muscle-function parameters, including appendicular skeletal muscle mass index (ASMI), grip strength, calf circumference, and 5-TCS performance (Zhang et al., 2025). Other beneficial taxa—*Phascolarctobacterium faecium, Faecalibacterium prausnitzii*, and *Fusicatenibacter saccharivorans*—are likewise significantly diminished (He et al., 2023). Notably, *F. prausnitzii* is a prominent butyrate producer (Jackson et al., 2016). Importantly, *P. faecium* abundance is positively associated with shikimate production, a precursor for aromatic amino acids that may modulate host protein synthesis (He et al., 2023). 2) Enrichment of potentially detrimental taxa: the relative abundances of *Phascolarctobacterium succinatutens, Clostridium* sp. *CAG 242, Clostridium* sp. *CAG 58*, and *Escherichia coli* are significantly elevated (He et al., 2023; Zhang et al., 2025). *P. faecium* is inversely correlated with *P. succinatutens*, suggesting that *P. faecium* may exert a growth-inhibitory effect on *P. succinatutens* (He et al., 2023).

In addition, Guan et al. reported that at the family level, *Tannerellaceae*, *unclassified_k:norank_d:Bacteria*, and *Eubacteriaceae* were significantly depleted in sarcopenic individuals (Guan et al., 2023). Notably, the butyrate-producing family *Eubacteriaceae* was markedly reduced (Louis and Flint, 2009; Vital et al., 2014; Guan et al., 2023), likely representing a key contributor to the observed decline in butyrate levels. LEfSe analysis (LDA > 3.0) further identified *Erysipelatoclostridium* as the most discriminative genus between sarcopenic and non-sarcopenic individuals (He et al., 2023).

Guo et al. performed 16S rDNA amplicon sequencing on fecal samples from older adults with low HGS and age-matched controls with normal HGS. Their results revealed a significant increase in the relative abundances of *Parabacteroides* and *Intestinibacter* in the low-HGS cohort. Moreover, the abundances of both *Parabacteroides* and *Intestinibacter* were inversely correlated with serum levels of cinnamoylglycine. Subsequent untargeted metabolomics further demonstrated markedly lower levels of cinnamic acid and its derivatives in the low-HGS group. Notably, six metabolites—including cinnamoylglycine—exhibited significant positive correlations with grip-strength values (Guo et al., 2024).

#### 2.4.2 Aberrant microbial metabolite regulation of muscle metabolic pathways

##### 2.4.2.1 Short-chain fatty acid deficiency impairs myoblast proliferation

Guan et al. reported that fecal butyrate—a key short-chain fatty acid—concentrations were significantly reduced in sarcopenic individuals. Moreover, butyrate correlated negatively with age and positively with both skeletal muscle mass and muscle function (Guan et al., 2023).

*In vitro* experiments revealed that 750 μM butyrate markedly enhanced C2C12 myoblast proliferation, an effect likely mediated by activation of the ERK/MAPK signaling axis, as evidenced by increased ERK1/2 phosphorylation and up-regulated mRNA and protein expression of the myogenic regulators *Myf5* and *MyoD* (Guan et al., 2023). Nevertheless, the precise role of Myf5 in regulating myoblast proliferation remains to be definitively established. It is noteworthy that prior studies have demonstrated expression of the short-chain fatty acid receptors GPR41 and GPR43 in muscle cells (Brown et al., 2003; Frampton et al., 2020); however, the contribution of these receptors was not directly examined in the present study through receptor knockdown or antagonist experiments. Furthermore, transcriptomic analyses revealed significant enrichment of PI3K–AKT pathway–related genes, suggesting that this pathway may synergistically contribute to butyrate-mediated proliferation—an observation warranting further investigation.

These findings indicate that butyrate exerts a dual—direct and indirect—protective effect on muscle health. In addition to directly modulating myocyte proliferation, its role in maintaining intestinal homeostasis cannot be overlooked. This notion is strongly supported by the work of Herrmetier et al.: In a mouse model of combined alcohol and burn injury, they observed marked gut dysbiosis and a significant drop in butyrate levels, and demonstrated that this directly exacerbated intestinal barrier injury and local inflammation (e.g., elevated IL-6) (Herrnreiter et al., 2025). Given that chronic systemic low-grade inflammation has been identified as a core pathological pillar driving muscle wasting (see Section 2.3), this evidence strongly suggests that butyrate deficiency may, via the indirect route of amplifying gut-derived inflammation, synergise with its direct effects on myocytes to constitute a network-level mechanism underlying gut–muscle axis dysfunction.

##### 2.4.2.2 Gut microbiota-niacin-NAD^+^ axis dysfunction impairs muscle regeneration and mitochondrial homeostasis

Metagenomic profiling of fecal samples from 80 participants by Zhang et al. revealed that individuals with sarcopenia exhibited significantly reduced serum levels of the beneficial metabolites nicotinamide mononucleotide (NMN), indole-3-lactate (ILA), indole-3-acetate (IAA), and nicotinic acid (NA). Random forest analysis further identified NA as the most discriminatory metabolite between sarcopenic and healthy individuals; its concentration correlated positively with multiple muscle function indices, including ASMI, grip strength, calf circumference, and 5TCS performance (Zhang et al., 2025).

Mechanistic studies confirmed that the nicotinic acid phosphoribosyltransferase (k00763), specifically encoded by *B. adolescentis*, catalyzes the biosynthesis of NA. Experiments in aged mice demonstrated that supplementation with either *B. adolescentis* or NA alone markedly increased the abundance of PAX7^+^ muscle stem cells (MuSCs) and the proportion of dystrophin-positive myofibers, concomitant with a significant elevation in tissue NAD^+^ levels. Functional annotation of metagenomic data revealed that the activities of five key pathways governing NAD^+^ metabolism were significantly down-regulated in sarcopenic individuals. Functional analyses further demonstrated that *B. adolescentis*/NA, via NAD^+^ enrichment, activates the SIRT1/PGC-1α axis, up-regulates NRF1, NRF2, and TFAM, increases the mtDNA/nuclear DNA ratio, and enhances mitochondrial biogenesis—as detailed in Section 2.2.4. Simultaneously, this axis suppresses the FoXO3/Atrogin-1/MuRF-1 pathway, thereby promoting MuSC proliferation. Collectively, these findings indicate that NA derived from *B. adolescentis* comprehensively ameliorates aging-related impairment of skeletal muscle function by elevating NAD^+^ levels (Zhang et al., 2025).

Collectively, the gut microbiota modulates host NAD^+^ homeostasis via the gut–muscle axis, thereby influencing skeletal-muscle mitochondrial function. Although previous studies employing FoxO1/3/4 triple-knockout and Atrogin-1-deficient cells have established the critical role of the FoxO3–Atrogin-1/MuRF-1 axis in acute muscle atrophy (Zhao et al., 2007; Milan et al., 2015), its causal relevance in naturally occurring chronic sarcopenia remains undefined. Future work should therefore employ muscle-specific conditional knockouts of FoxO3 or Atrogin-1 in aged sarcopenic mouse models to determine whether disruption of this pathway can reverse age-related functional decline and to clarify its precise contribution to chronic muscle wasting.

##### 2.4.2.3 Gut microbiota-bile acid axis disruption in mitochondrial toxicity and signaling interference

Bile acids, cholesterol-derived steroids secreted by the liver, are essential for the digestion and absorption of dietary lipids. In the pathogenesis of sarcopenia, microbiota-mediated disruption of bile-acid metabolism has emerged as a novel disease-promoting hypothesis. Specifically, levels of taurine-conjugated bile acids that positively correlate with skeletal-muscle index and grip strength—e.g., taurocholic acid (TCA) and taurolithocholic acid (TLCA)—are decreased, whereas concentrations of unconjugated acids that inversely associate with muscle loss—e.g., cholic acid (CA), chenodeoxycholic acid (CDCA), and ursodeoxycholic acid (UDCA)—are increased (Marques et al., 2023). At high concentrations, these bile acids exert direct mitochondrial toxicity, triggering the mitochondrial permeability transition (MPT) characterized by impaired electron-transport-chain activity, diminished respiration, mitochondrial swelling, and increased outer-membrane permeability, events that can culminate in the initiation of programmed cell death (Krähenbühl et al., 1994; Rolo et al., 2000; Palmeira and Rolo, 2004). This cascade is primarily driven by oxidative stress and remains partially responsive to antioxidant intervention (Sokol et al., 2001).

Moreover, gut dysbiosis—exemplified by decreased bile-salt hydrolase (BSH) activity—distorts the circulating bile-acid pool, leading to accumulation of farnesoid X receptor (FXR) antagonists, such as tauro-β-muricholic acid (TβMCA). This suppresses intestinal FXR signaling and diminishes the synthesis and release of its downstream endocrine hormone, fibroblast growth factor 15/19 (FGF15/19) (Li et al., 2013; Sayin Sama et al., 2013; Qiu et al., 2021). Reduced circulating FGF15/19 has been shown to impair anabolic signaling cascades—including ERK/mTOR—in skeletal muscle (Benoît et al., 2017) and to up-regulate the expression of atrogenes such as Atrogin-1 and MuRF-1 (Lahiri et al., 2019; Mancin et al., 2023). Functional studies in animal models have validated this axis, and preliminary human data reveal significant associations among fecal microbiota composition, plasma FGF19 levels, and muscle mass in older adults and individuals with sarcopenia (Qiu et al., 2022). Nevertheless, owing to intrinsic interspecies differences in bile acid profiles and FGF signaling between humans and mice, the causal relevance and therapeutic potential of this pathway in human sarcopenia must be definitively established through more extensive clinical investigations.

Collectively, bile acids contribute to the initiation and progression of sarcopenia through multiple mechanisms, including mitochondrial dysfunction and disruption of the FXR–FGF15 endocrine axis. These findings establish a theoretical framework positioning bile acids as potential biomarkers and therapeutic targets for sarcopenia, while also providing clear directions for future mechanistic investigations and translational research.

##### 2.4.2.4 Aberrant carbohydrate-metabolizing enzymes underlie energy insufficiency

Shotgun metagenomic sequencing and untargeted metabolomic profiling of blood and fecal samples from 63 older adults in Haikou, China—including both sarcopenic patients and healthy controls—revealed marked alterations in amino-acid metabolic pathways of the gut microbiota of sarcopenic individuals, together with aberrant expression of genes encoding carbohydrate-active enzymes (CAZymes), indicating impaired capacity for complex carbohydrate metabolism. At the metabolite level, fecal concentrations of shikimate—a precursor of aromatic amino acids—and several bioactive di- and tri-peptides (e.g., Tyr-Ala, Pro-Gly-Asn) were significantly lower in sarcopenic subjects. Further analyses revealed that *Phascolarctobacterium faecium* enhances shikimic acid production, thereby modulating the biosynthesis of phenylalanine, tyrosine, and tryptophan (He et al., 2023). Previous studies have demonstrated that phenylalanine, tyrosine, and tryptophan not only directly stimulate skeletal-muscle protein synthesis but also modulate muscle mass (Volpi et al., 2003; Ryan et al., 2008). Consequently, the observed decline in the abundance of *Phascolarctobacterium faecium* may impair muscle-protein synthesis signaling indirectly—via disruption of these aromatic amino-acid pathways—and ultimately compromise skeletal-muscle mass. This finding offers a novel perspective on the gut–muscle axis.

In summary, this review delineates characteristic dysbiosis in sarcopenic patients: a decline in beneficial taxa—especially SCFA producers—coupled with an expansion of potentially harmful species that collectively disrupt carbohydrate metabolism and diminish beneficial metabolites, such as shikimate (He et al., 2023). These findings provide a theoretical foundation for developing gut-microbiota-targeted interventions against sarcopenia.

#### 2.4.3 Microbiota-targeted interventions show therapeutic promise

Thus, converging evidence indicates that disruption of the gut–muscle axis is a key driver of sarcopenia, operating through multi-layered biological pathways. At the compositional level, this disruption is characterized by a marked depletion of beneficial taxa, such as *Bifidobacterium adolescentis*, and a parallel overgrowth of potentially pathogenic species, exemplified by Escherichia coli. Such dysbiosis directly curtails SCFA production, thereby perturbing NAD^+^ metabolic homeostasis, deranging aromatic amino-acid biosynthesis, and mediating bile-acid metabolic chaos. Molecularly, these alterations converge on inhibition of the ERK/MAPK signaling cascade, impairment of the SIRT1/PGC-1α functional axis, and activation of FoxO3-driven proteolytic pathways, collectively precipitating the progressive decline in muscle mass and function.

These findings open new avenues for the prevention and treatment of sarcopenia, including 1) the screening and application of specific functional strains, 2) supplementation of key metabolites, and 3) targeted modulation of relevant signaling pathways. Nevertheless, several critical issues remain to be addressed, such as the influence of population heterogeneity and the need for experimental validation of causal relationships. Future research should focus on developing more robust animal models and accelerating the translation of fundamental findings into clinical applications.

### 2.5 Systemic synergistic factors: hormones and nutritional metabolism

#### 2.5.1 Declining insulin-like growth factor-I (IGF-1) impairs mitochondrial protection

IGF-I plays a pivotal role in skeletal-muscle growth and repair. Compared with non-sarcopenic controls, sarcopenic individuals exhibited lower circulating IGF-I levels and a reduced IGF-I/IGF-binding protein 3 (IGFBP3) ratio, although these differences did not reach statistical significance. Furthermore, IGF-I levels were inversely correlated with three very-long-chain fatty acids (VLC-FAs: NEFA 26:2, 24:4, and 24:2) and with Cit; similarly, the IGF-I/IGFBP3 ratio showed negative associations with multiple long- and very-long-chain non-esterified fatty acids (NEFAs) (Marques et al., 2023). These observations suggest that IGF-I is implicated in the metabolic pathways underlying sarcopenia.

Mediation analysis further revealed that IGF-I exerts a significant indirect effect on sarcopenia by modulating VLC-FA (Marques et al., 2023). In addition, low circulating IGF-I levels are associated with mitochondrial dysfunction in aged rats, manifesting as increased mitochondrial permeability, loss of membrane potential, elevated proton leak, excessive free-radical generation, and reduced activities of ATP synthase and complex IV. Exogenous IGF-I administration markedly ameliorates these impairments, attenuates oxidative stress damage, and restores antioxidant enzyme activities in ageing mice (García-Fernández et al., 2008; Puche et al., 2008; Sádaba et al., 2016). Collectively, these data establish IGF-I as a cytoprotective factor that preserves mitochondrial integrity, limits free-radical production, mitigates oxidative injury and apoptosis, and enhances ATP generation (Puche et al., 2008). With advancing age, circulating growth hormone and plasma IGF-I levels decline in both humans and animal models, further supporting the involvement of IGF-I in sarcopenia (Sonntag et al., 1980; Florini et al., 1981; Ferrari et al., 2021). These findings indicate that the age-related reduction in IGF-I may indirectly contribute to the development of sarcopenia by disrupting fatty-acid metabolism and impairing mitochondrial function. Future investigations should delineate the precise molecular mechanisms through which IGF-I influences sarcopenia and rigorously evaluate its therapeutic potential, thereby offering novel strategies for the clinical management of the disease.

In summary, given the current paucity of omics-level investigations into the role of the IGF system in sarcopenia, the mechanisms discussed in this section are largely derived from conventional experimental approaches. To overcome this limitation, future studies must adopt integrated multi-omics frameworks that jointly analyze genomic, epigenomic, transcriptomic, proteomic, and metabolomic datasets to construct comprehensive molecular-regulatory networks, thereby illuminating IGF-centric synergistic pathomechanisms on a global scale. Furthermore, deploying single-cell and spatially resolved technologies to dissect cell-type-specific alterations of IGF signaling in myofibers, satellite cells, immune cells, and other populations will greatly refine our understanding of sarcopenia heterogeneity and unveil novel targets for precision intervention.

#### 2.5.2 Nutritional and metabolic perturbations exacerbate sarcopenia via multiple mechanisms

Lu et al. analyzed blood samples from 189 community-dwelling older adults to investigate the associations among nutritional and metabolic biomarkers, gene expression, and sarcopenia (Lu et al., 2020). After adjustment for age and sex, Mini Nutritional Assessment (MNA) scores and risk of malnutrition were independently associated with the presence of sarcopenia. These findings indicate that nutritional status is an important determinant of sarcopenia. Specifically, nutritional status, as measured by the MNA score, was primarily associated with muscle mass preservation and, to a lesser extent, with muscle strength, highlighting the essential role of nutrition in maintaining muscle health (Hai et al., 2017).

Regarding metabolic biomarkers, body mass index (BMI) and leptin showed inverse associations with sarcopenia, whereas adiponectin and high-density lipoprotein (HDL) showed positive correlations. These findings suggest that global metabolic status may influence the development of sarcopenia through multiple pathways. Moreover, sarcopenia was inversely and significantly associated with circulating levels of several EAAs, including lysine, methionine, phenylalanine, and threonine, as well as BCAAs and choline. These EAAs are indispensable for muscle protein synthesis; their depletion may attenuate anabolic capacity, thereby facilitating the onset of sarcopenia.

Emerging evidence has reshaped our understanding of the relationship between vitamin D and sarcopenia. A cross-sectional study of 368 rural Chinese adults aged ≥ 65 years revealed that, among individuals with sufficient vitamin D (serum 25-hydroxyvitamin D_3_ (25(OH)D_3_) > 20 ng mL^-^¹), vitamin D status was not associated with sarcopenia or its components (grip strength, gait speed, or SMI); in the vitamin-D-deficient subgroup (< 12 ng mL^-^¹), supplementation elicited only a modest improvement in grip strength (Mi et al., 2025). This observation is consistent with a systematic review encompassing 35 trials (6,628 participants), which demonstrated that oral vitamin D, even at high doses, produced no significant improvements in appendicular skeletal muscle mass, grip strength, or Timed Up-and-Go (TUG) performance, with an overall negligible effect size (Widajanti et al., 2024). Notably, Lu and colleagues uncovered a potential mechanism underlying impaired vitamin D signaling: although serum 25(OH)D_3_ levels did not differ between sarcopenic and non-sarcopenic older adults, the proto-oncogene serine/threonine-protein kinase *PIM1* was markedly down-regulated in sarcopenic individuals (Lu et al., 2020). Further mechanistic studies have revealed that the kinase PIM1 modulates downstream signaling by directly interacting with the vitamin D receptor’s DNA-binding domain. Collectively, these findings indicate that the efficacy of vitamin D intervention in sarcopenia may hinge on the integrity of its intracellular signaling axis rather than on circulating levels alone, offering a novel molecular perspective on sarcopenia pathogenesis.

In conclusion, future investigations should rigorously delineate and validate the specific molecular mechanisms underlying these factors in sarcopenia and critically evaluate their potential as diagnostic biomarkers and therapeutic targets, thereby informing innovative clinical strategies for sarcopenia management.

## 3 Disease-specific mechanisms of sarcopenia comorbid with chronic liver disease: multi-omics evidence and axial regulation

**Figure 2:**
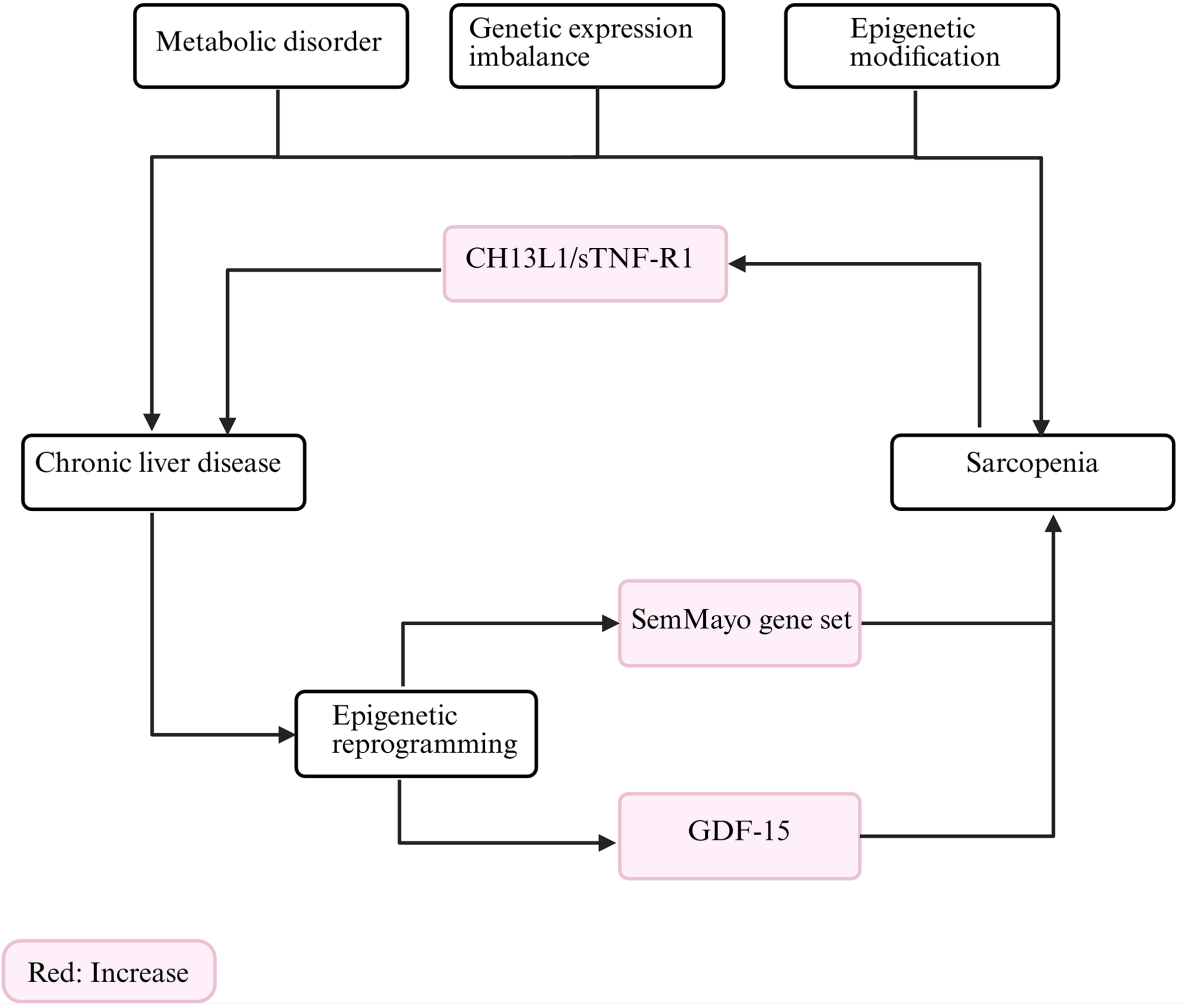
The muscle-liver axis in chronic liver diseases: A model of pathological interaction and malignant cycle. CHI3L1, Chitinase-3-Like Protein 1; sTNF-R1, Soluble Tumor Necrosis Factor Receptor 1; GDF-15, Growth Differentiation Factor 15. (Created in BioRender. HUI, X. (2026) https://BioRender.com/jirt514).

**Figure 3:**
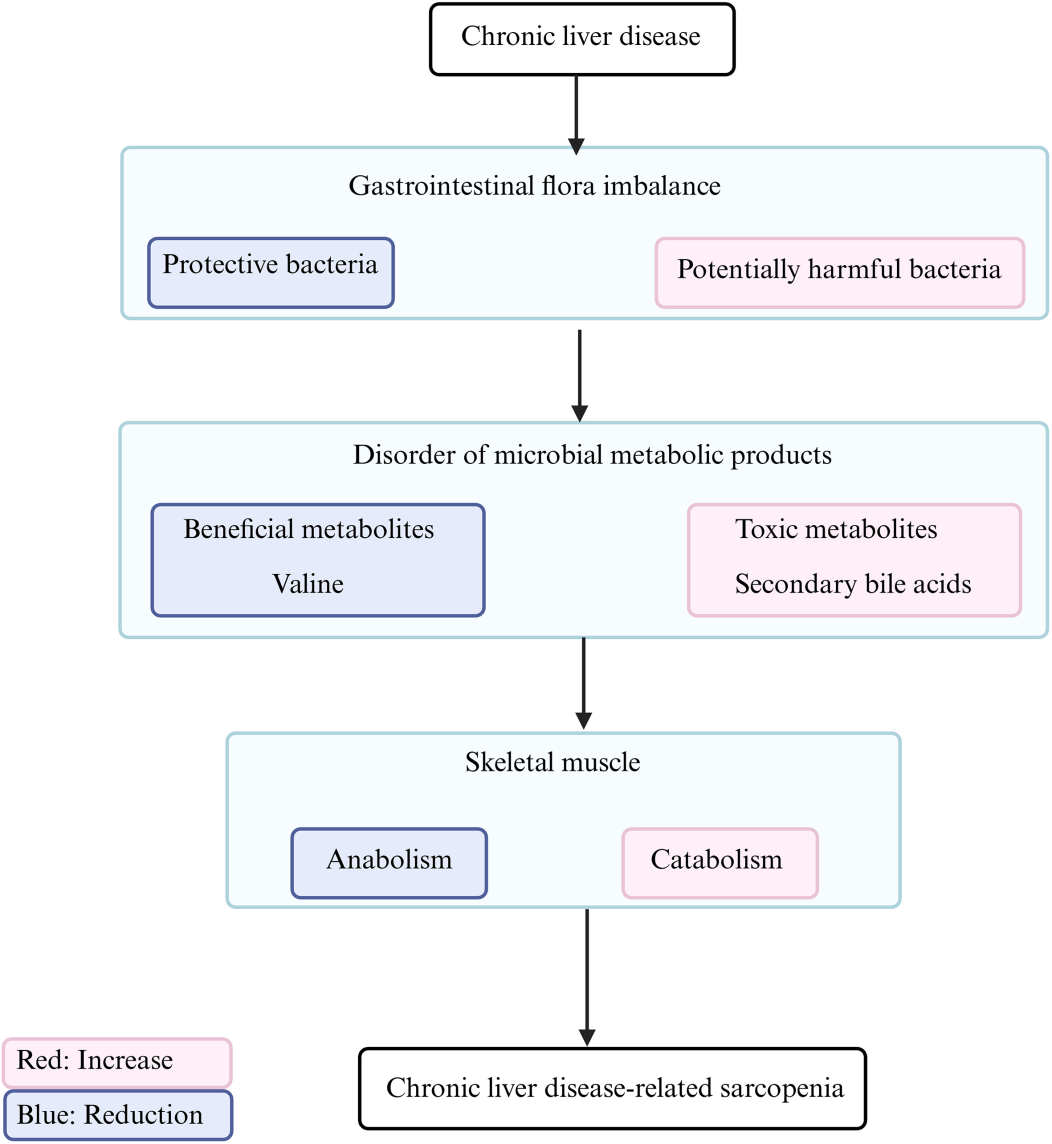
The gut-muscle axis remodeled by chronic liver disease: The liver-gut-muscle pathway. (Created in BioRender. HUI, X. (2026) https://BioRender.com/haamdel).

Building on the preceding systematic review of general sarcopenia mechanisms revealed by omics technologies, this chapter narrows the focus to the specific comorbid context of CLD. It delineates how CLD sculpts disease-specific pathobiology by remodeling the “muscle–liver axis” (**Figure 2**) and the “gut–muscle axis” (**Figure 3**), whose shared foundations were outlined in Chapter 2. The subsequent discussion will organize multi-omics evidence within this dual-axis theoretical framework to uncover the unique mechanisms by which the liver-disease microenvironment precipitates muscle wasting (**Table 3**; Details are summarized in **Supplementary Table S3**). The trans-omics evidence pertaining to the core pathological mechanisms of sarcopenia in the context of chronic liver disease has been synthesized and consolidated in **Supplementary Table S4**.

**Table 3:** Overview of core omics studies informing the multi-system molecular framework of sarcopenia associated with chronic liver disease.

Author	Year	Sample size	Types of omics analysis	Main findings
Irina Efremova	2024	40	Microbiome Analysis	The abundances of *Akkermansia* and *Eggerthella* are independent determinants of SMI.
Thomas Nicholson	2024	56	Epigenomics Analysis	CLD is associated with epigenetic and transcriptomic reprogramming in both skeletal muscle and the immune system, driving an accelerated aging phenotype. This inherent cellular aging may contribute to the development of sarcopenia and immune dysfunction in patients with CLD.
			Transcriptomics Analysis	
			Immunophenotyping	
Benard Aliwa	2023	175	Metabolomics Analysis	1.*Bacteroides ovatus* is associated with cirrhosis without sarcopenia and shows positive correlations with BMI, muscle mass, valine, and acetate
			Microbiomics Analysis	2.In sarcopenic patients with cirrhosis, gut microbiome–host interactions are associated with an altered gut microbiome composition, modified bile-acid profiles, and reduced serum valine, pointing toward a potential cirrhosis-specific mechanistic interplay in understanding the pathogenesis of sarcopenia in cirrhosis.
Ziying Xu	2023	170	Transcriptomics Analysis	The gene expression patterns of NAFLD and sarcopenia exhibit a significant positive correlation. We identified four pairs of genes that are co-up-regulated or co-down-regulated: *HIF1A, ATG5, ADM*, and *CST3* are highly expressed in both diseases, whereas *BMP2, BMPR2, TFDP1*, and *E2F6* are expressed at low levels.
Kenta Yamamoto	2022	69	Microbiome Analysis	Patients with low muscle mass in chronic liver disease had a significantly lower *Firmicutes* to *Bacteroidetes* ratio and may contain more gram-negative bacteria and more LPS. Their microbiome contains fewer enzymes that synthesize intestinal nitrogen into BCAAs.
Di Lu	2022	136	Transcriptomics Analysis	In patients with sarcopenia, CHI3L1 is upregulated and secreted by skeletal muscle via the TNF-α/TNFR1 signaling pathway, protecting myocytes from damage, while concurrently promoting HCC progression by inducing the accumulation of LPO products.
			Metabolomics Analysis	

CLD, Chronic liver disease; NAFLD, non-alcoholic fatty liver disease; LPO, Lipid peroxidation; HCC, Hepatocellular carcinoma; TNF-α, Tumor necrosis factor-alpha; TNF-R 1, Tumor necrosis factor receptor 1.

### 3.1 From crosstalk to vicious cycle: omics-driven dissection and pathological remodeling of the muscle–liver axis

#### 3.1.1 Common ground: physiological interactions and metabolic coupling of the muscle–liver axis

Emerging evidence indicates that the liver and skeletal muscle form a bidirectionally regulated “muscle–liver axis.” As a metabolic hub, the liver remotely governs muscle protein synthesis and energy metabolism by releasing hormones and metabolites such as FGF21 and IGF-1 (Holecek et al., 2011; Wing et al., 2011; Sanchis-Gomar et al., 2014; Bhanji et al., 2017; Yuan et al., 2024); conversely, muscle-derived myokines (e.g., myostatin) feedback to modulate hepatic glucose and lipid homeostasis (Nishikawa et al., 2017). This physiological crosstalk underpins whole-body metabolic equilibrium. Genome-wide association studies further demonstrate a significant positive genetic correlation between non-alcoholic fatty liver disease and both muscle mass and grip strength, implying a shared genetic architecture (Yuan et al., 2024). Collectively, these data establish the muscle–liver axis as a common, inter-organ metabolic nexus. In chronic liver disease, however, this axis is destabilized, and its regulatory network is profoundly remodeled. Notably, immunosuppressants used after liver transplantation to prevent rejection—such as calcineurin inhibitors (CNIs) and mTOR inhibitors (mTORis)—can, at a distance, drive systemic pathology by reshaping the immune microenvironment, offering a direct pathophysiological paradigm for understanding how liver disease systemically drives muscle wasting (Chen et al., 2024).

#### 3.1.2 Disease-specific exacerbation: pathological remodeling of the muscle–liver axis in chronic liver disease

##### 3.1.2.1 Metabolic–epigenetic co-regulation: systemic disruption of the muscle–liver axis

By integrating transcriptomic datasets from patients with sarcopenia and non-alcoholic fatty liver disease (NAFLD), Xu et al. employed multidimensional bioinformatic analyses to systematically dissect the molecular features shared by both disorders (Xu et al., 2023). Functional enrichment of the top 1,000 most highly expressed genes revealed significant overlap across several key metabolic pathways, notably lipid metabolism (e.g., fatty acid β-oxidation and cholesterol metabolism), oxidative stress (including ROS pathways and antioxidant responses), and energy metabolism (e.g., the TCA cycle and mitochondrial function). Gene-set variation analysis (GSVA) further showed that purine nucleotide salvage and lipoxygenase pathways were markedly associated with both conditions. Detailed interrogation demonstrated that the shared genes are principally involved in ribonucleoprotein-complex biogenesis, ribosome assembly, non-coding RNA processing, histone modification, and rRNA metabolism.

Further analyses revealed a significant positive correlation in gene-expression patterns between NAFLD and sarcopenia. Leveraging these shared signatures, the authors constructed a pathway-gene functional network. This approach identified four pairs of synergistically regulated hub genes: *HIF1A, ATG5, ADM*, and *CST3* were consistently upregulated, whereas *BMP2, BMPR2, TFDP1*, and *E2F6* were down-regulated in both disorders. These hub genes exert critical regulatory functions in both liver and skeletal muscle; for example, *HIF1A* modulates hepatic lipogenesis while also being intimately involved in muscle atrophy, exemplifying the molecular inter-organ crosstalk characteristic of the muscle–liver axis (Xu et al., 2023).

The transcriptomic findings described above are corroborated by the genome-wide association study (GWAS) conducted by Yuan et al. Using LD-score regression (LDSC), they demonstrated a significant positive genetic correlation between NAFLD and both grip strength and appendicular lean mass (ALM), implying a partial overlap in their genetic architecture. Gene-level analyses identified 153 genes significantly associated with both NAFLD and ALM, 24 of which also influenced grip strength. GTEx expression profiles revealed that these genes are enriched in liver, skeletal muscle, pancreas, blood, and brain, and are over-represented in pathways related to antigen presentation, cytokine signaling, and mitochondrial function (Yuan et al., 2024).

Collectively, the data demonstrate that the comorbidity between sarcopenia and NAFLD is orchestrated by a complex regulatory network integrating metabolic perturbations, transcriptional dysregulation, and epigenetic modifications. These findings not only validate the bidirectional control hypothesis of the muscle–liver axis, but also raise pivotal questions: 1) How do epigenetic mechanisms spatio-temporally regulate axis-critical genes? 2) Do these epigenetic marks undergo dynamic stage-specific changes during disease progression? 3) Can novel interventional strategies be designed to target these epigenetic signatures? Answering these questions will propel muscle–liver-axis research from mechanistic exploration to clinical translation.

##### 3.1.2.2 The CHI3L1–LPO axis: a myogenic signal driving the vicious cycle of liver disease

In chronic liver disease, especially HCC, muscle–liver-axis interactions acquire distinct pathological features. A study of 136 male HCC patients undergoing liver transplantation revealed that skeletal-muscle-derived chitinase-3-like protein 1 (CHI3L1) and its soluble receptor sTNF-R1 are markedly elevated and closely linked to lipid-metabolic derangement, identifying them as key molecules associated with sarcopenia and predicting HCC recurrence (Lu et al., 2022). A murine model of severe cachexia further confirmed that CHI3L1 concentrations rose in parallel in atrophied skeletal muscle and peripheral blood, underscoring its potential as an inter-organ signaling molecule. Mechanistically, inflammatory stimuli upregulate CHI3L1 expression in myocytes via the tumor necrosis factor-alpha (TNF-α)/TNF receptor 1 (TNF-R1) axis, initially conferring transient cytoprotection. However, once CHI3L1 is abundantly secreted into the circulation, it exerts a diametrically opposite, tumor-promoting effect in the liver. Transcriptomic profiling of tumor tissue revealed that elevated plasma CHI3L1 levels were positively correlated with aberrant PPAR signaling, peroxisomal function, and arachidonic-acid metabolism. *In vitro* co-culture assays demonstrated that myocyte-secreted CHI3L1 drives lipid peroxidation (LPO) accumulation, thereby markedly enhancing the invasive capacity of Hep1–6 cells. Antioxidant α-tocopherol reversed this CHI3L1-mediated pro-invasive effect by suppressing LPO. More importantly, the study delineates a “vicious cycle” within the muscle–liver axis in chronic liver disease: the tumor microenvironment persistently releases inflammatory factors that exacerbate muscle atrophy and augment CHI3L1 secretion; in turn, elevated circulating CHI3L1 reprograms hepatic metabolism via lipid peroxidation, accelerating tumor invasion. This pathway reveals that atrophying muscle is no longer merely a metabolic victim, but rather an active contributor that drives malignant disease progression in the context of liver pathology.

##### 3.1.2.3 Systemic senescence: chronic liver disease drives remote effects via the muscle–liver axis

Emerging evidence indicates that CLD elicits systemic epigenetic reprogramming, thereby accelerating ageing across multiple tissues. Using an epigenetic-clock approach, Nicholson et al. observed that skeletal muscle in CLD patients exhibits a significantly older epigenetic age than in healthy controls (2.2 ± 4.8 years vs. −3.0 ± 3.2 years) (Nicholson et al., 2024). This phenomenon was particularly pronounced in the ALD and NAFLD subgroups. Such epigenetic alterations correlated positively with quadriceps intramuscular adipose tissue (IMAT) and dry BMI, and were accompanied by a 5.2-fold increase in the senescence marker Growth Differentiation Factor 15 (GDF-15), implicating metabolic disturbance as a key driver of this process. Subsequent analyses revealed significant enrichment of senescence-associated genes (SemMayo gene set) in CLD skeletal muscle, providing molecular evidence that cellular senescence is accelerated in these patients’ skeletal muscle.

Importantly, these epigenetic anomalies are not restricted to skeletal muscle; a parallel acceleration is observed in peripheral blood mononuclear cells (PBMCs) and correlates significantly with the degree of muscle ageing. Immunologically, patients with CLD display classic immune-senescence signatures: an aberrantly high proportion of senescent CD8^+^ T cells, exhausted CD4^+^T cells and regulatory T cells, coupled with a reduction in class-switched memory B cells (IgD^-^CD27^+^) and memory B cells (CD24^high^ CD38^−^). Of particular note, the IMM-AGE score—a composite metric of immune ageing—was twice as high in CLD patients as in healthy controls and correlated significantly with indices of declining muscle function, such as grip strength (Nicholson et al., 2024).

Collectively, these findings delineate a systemic aging paradigm triggered by chronic liver disease: hepatic pathology—via epigenetic reprogramming, a chronically inflamed microenvironment, and metabolic derangement—propagates through the muscle–liver axis, culminating in muscle atrophy and immune dysfunction. This mechanism not only deepens our understanding of the systemic consequences of CLD but also furnishes a theoretical rationale and potential biomarkers for novel interventions—such as senolytic therapies or epigenetic modulators. In particular, metrics like GDF-15 and the IMM-AGE score may serve as key benchmarks for monitoring disease progression and therapeutic efficacy.

In summary, this study employed multi-omics integration to provide an initial characterization of how the “muscle–liver axis” drives sarcopenia specifically within the context of CLD. Unlike simple metabolic associations, the muscle–liver axis in CLD exhibits hallmark features of systemic disruption, vicious-cycle amplification, and accelerated senescence. It must be emphasized, however, that omics investigations dedicated to this comorbid setting remain scarce; consequently, the mechanisms outlined herein represent only fragments of a far more intricate pathological network. Future efforts should urgently deploy cutting-edge technologies, such as spatial omics and single-cell multi-omics, to conduct deeper, more systematic analyses within the “commonality–specificity” framework, ultimately unveiling the full network-level regulatory architecture of the muscle–liver axis in CLD-associated sarcopenia.

### 3.2 From micro-ecology to multi-dimensional networks: distinct disruption and integration models of the gut–muscle axis

As previously discussed, gut dysbiosis collectively promotes sarcopenia by reducing SCFA synthesis, perturbing aromatic amino acid biosynthesis, and disrupting bile acid metabolism. At the molecular level, these alterations converge to inhibit the ERK/MAPK cascade, impair the SIRT1/PGC-1α functional axis, and activate FoxO3-driven proteolytic pathways, resulting in progressive loss of muscle mass and function. Nevertheless, within the specific pathological milieu of CLD, the precise contribution of the gut–muscle axis to sarcopenia development remains incompletely defined and warrants in-depth investigation. Recent advances in omics technologies now provide powerful tools to systematically dissect the mechanisms underlying the gut–muscle axis in CLD-associated sarcopenia.

Against this background, the present chapter integrates data from three independent studies (Yamamoto et al., 2022; Aliwa et al., 2023; Efremova et al., 2024) to systematically analyze 16S rDNA/rRNA sequencing, metabolomic profiles, and functional-prediction outputs. To this end, we first delineate the gut-microbial and metabolic signatures that distinguish CLD patients with sarcopenia from those without sarcopenia. Subsequently, by interpreting these signatures within CLD-specific pathophysiological contexts—such as portal hypertension and hepatic dysfunction—we aim to reveal how chronic liver disease acts as a predisposing driver that actively sculpts and exacerbates a distinct pattern of gut–muscle axis disruption. This pathophysiological cascade, initiated by hepatic pathology, mediated by gut microbiota and their metabolites, and ultimately targeting skeletal muscle, constitutes the core concept of the “liver–gut–muscle axis.

#### 3.2.1 Common ground: core disruption of the gut–muscle axis in sarcopenia

As outlined in Section 2.4, the onset and progression of sarcopenia are underpinned by a core gut–muscle–axis disruption that is conserved across distinct etiologies. Multi-omics integration by Aliwa et al. provides direct evidence: irrespective of cirrhosis status, sarcopenic patients exhibit diminished microbial fatty acid biosynthesis and impaired pathways involved in genetic information processing and cell division (Efremova et al., 2024). Concordantly, the same study revealed marked enrichment of BCAA catabolic pathways at the host level, indicating that systemic BCAA metabolic derangement is a shared sarcopenic trait. Beneath this common pattern, however, lies profound mechanistic complexity. Notably, Zuo et al. painted an opposite picture at the myofiber level: deficiencies in BCAA-catabolic enzymes (BCAT2, BCKDHB) cause local BCAA accumulation and sustained downstream mTOR signaling in sarcopenic muscle (Zuo et al., 2025). This exposes a central paradox: while systemic BCAA catabolism is globally up-regulated, skeletal muscle exhibits local BCAA utilization failure and sequestration. The coexistence of “systemic depletion” and “local pooling” underscores the intricate regulation of the gut–muscle axis and implies that intrinsic myocellular metabolic defects, together with microbiota-driven systemic metabolic states, cooperatively exacerbate disease progression.

#### 3.2.2 Disease-specific exacerbation: unique gut-muscle axis disruption sculpted by CLD

##### 3.2.2.1 Microbial ecosystem deterioration in the context of chronic liver disease

Numerous recent studies have sought to clarify the link between gut microbiota and liver-disease-associated sarcopenia; however, conclusions remain inconsistent regarding the crucial issue of microbial diversity changes.

Efremova et al. found no significant differences in overall gut microbial diversity or in the abundance of dominant taxa—the four most abundant phyla and the three largest orders within Bacillota—between cirrhotic patients with sarcopenia and those without sarcopenia (Efremova et al., 2024). In contrast, among minor microbial taxa, skeletal-muscle mass in cirrhotic-sarcopenic patients correlated with the abundance of several bacterial groups, most notably Akkermansia and Eggerthella. However, the study identified associations only and did not establish causality. SMI was positively associated with Akkermansia abundance and negatively associated with Eggerthella abundance. Moreover, the abundances of Akkermansia and Eggerthella were independent determinants of SMI (Efremova et al., 2024). These findings indicate that specific bacterial genera—rather than overall community structure—are key to discriminating sarcopenia in this population.

Building on these observations, Aliwa et al. conducted a more comprehensive characterization of the sarcopenia-specific microbiota in patients with liver cirrhosis (Aliwa et al., 2023). Their findings likewise confirmed that, although gut microbial α- and β-diversity did not differ between groups, sarcopenic patients harbored a distinct community structure. Relative abundances of Bacteroides fragilis, Blautia marseille, Sutterella spp., and Veillonella parvula were significantly increased and all inversely correlated with BMI, muscle mass, and mid-arm muscle circumference (MAMC). Conversely, Bacteroides ovatus was enriched in non-sarcopenic patients; its abundance positively correlated with muscle mass, MAMC, and serum valine and acetate levels. Most importantly, multivariate regression identified B. ovatus as an independent protective predictor of sarcopenia, an association that remained robust after adjustment for medication use and liver-disease severity (Aliwa et al., 2023).

In contrast, Yamamoto and colleagues stratified chronic liver disease patients solely by SMI into low-SMI (L-SMI) and normal-SMI (N-SMI) groups, and reported that α-diversity was significantly lower in the L-SMI cohort, indicating reduced intestinal microbial richness (Yamamoto et al., 2022). Consistently, β-diversity did not differ between the two groups, suggesting that the overall microbial community structure was broadly similar. At the phylum level, the relative abundances of *Proteobacteria* and *Bacteroidetes* were higher in the L-SMI group, whereas *Firmicutes* abundance and the *Firmicutes*/*Bacteroidetes* ratio were lower compared with the N-SMI group. Genus-level analyses further showed that *Coprobacillus*, *Catenibacterium*, and *Clostridium* were significantly less abundant, while *Bacteroides* was more abundant in the L-SMI group, with all differences achieving statistical significance (Yamamoto et al., 2022). Comparison with previous findings suggests that sarcopenia defined by SMI alone versus by comprehensive diagnostic criteria may capture distinct α-diversity states, offering a potential explanation for the inconsistent conclusions across studies.

In summary, despite inconsistent findings regarding α-diversity, multiple studies converge on a structural microbiota signature of sarcopenia in chronic liver disease: depletion of protective genera such as *Akkermansia* and expansion of opportunistic pathogens, including *Eggerthella* and Bacteroides fragilis. This distinct ecological configuration is likely sculpted by the intestinal milieu characteristic of liver disease—e.g., portal hypertension and altered bile-acid pools—and provides a microbial basis that differentiates these patients from those with stable liver disease or from patients with non-hepatic sarcopenia.

##### 3.2.2.2 Microbial functional alterations and metabolic disturbances in CLD

Efremova and colleagues found that, compared with cirrhotic patients without sarcopenia, those with sarcopenia harbored a gut microbiota enriched in genes involved in lipopolysaccharide biosynthesis, carbohydrate digestion and absorption, folate biosynthesis, and the citrate cycle, but depleted in genes related to antibiotic biosynthesis, biofilm formation, and the pentose-phosphate pathway (Efremova et al., 2024). Integrating prior data, *Akkermansia* is thought to preserve muscle mass by reinforcing intestinal barrier function and suppressing lipopolysaccharide (LPS) translocation and ensuing inflammation (Everard et al., 2013). Conversely, *Eggerthella* may exacerbate muscle catabolism by activating Th17 inflammatory pathways and enhancing secondary bile-acid metabolism (Wegner et al., 2017; Alexander et al., 2022). Collectively, insufficient *Akkermansia* and excessive *Eggerthella* abundances appear to play important roles in the development of sarcopenia in liver cirrhosis, although their precise mechanisms remain to be fully elucidated (Efremova et al., 2024).

At the level of bile acid metabolism, Aliwa et al. demonstrated characteristic dysregulation in cirrhotic patients with sarcopenia (Aliwa et al., 2023). Compared with their non-sarcopenic counterparts, these individuals exhibited markedly higher serum concentrations of secondary bile acids (total DCA, total LCA, unconjugated DCA, and unconjugated LCA) and greater fecal total LCA levels. In parallel, several indices reflecting the conversion of primary to secondary bile acids—namely serum DCA: CA, LCA: CDCA and fecal DCA: CA, LCA: CDCA ratios—were significantly elevated. Collectively, these shifts implicate enhanced microbial 7α-dehydroxylation activity as the central driver. Multivariate analyses further identified distinct bile-acid profiles—exemplified by the serum 12-α-OH:non-12-α-OH BA ratio and the T-UDCA:total-secondary-BA ratio—as independent predictors of sarcopenia, underscoring their pathogenic significance (Aliwa et al., 2023).

Although metagenomic profiling revealed no statistically significant differences in the relative abundance of bile-acid-metabolizing functional genes—such as bile-salt hydrolase (BSH) and 7α-hydroxysteroid dehydrogenase (7α-HSDH)—between sarcopenic and non-sarcopenic individuals, post-transcriptional regulation or microbial synergism may still modulate gene expression and enzymatic activity. Previous studies have demonstrated that, in hypercholanemic conditions typical of chronic liver disease, elevated bile acids (e.g., DCA, CA) activate the TGR5 receptor on skeletal muscle cell membranes. Such activation triggers intracellular oxidative stress and up-regulates muscle-specific E3 ubiquitin ligases (e.g., Atrogin-1 and MuRF-1), thereby stimulating proteolytic pathways including the ubiquitin–proteasome system and autophagy, ultimately culminating in myofiber atrophy and sarcopenia (Abrigo et al., 2021). More notably, an imbalanced bile-acid profile can also precipitate liver injury through multiple interacting mechanisms—direct cytotoxicity, oxidative stress, inflammatory cascades, and disruption of signaling networks (Li et al., 2024). Therefore, future work should integrate *in vitro* cellular models (e.g., human skeletal-muscle cell lines) and animal models to definitively validate the regulatory effects of specific bacterial taxa and their metabolites—such as DCA and LCA—on muscle biology (Aliwa et al., 2023).

In addition, the research team employed comprehensive metabolomic profiling and found that cirrhotic patients with sarcopenia exhibited significantly lower abundance of *Bacteroides ovatus* in their gut microbiota than those without sarcopenia, accompanied by a marked decrease in serum valine levels. Further analyses revealed that serum valine concentrations were positively correlated with key sarcopenia-related indicators, including BMI, muscle mass, and mid-arm muscle circumference. Multivariate regression analysis further identified serum valine as an independent predictor of sarcopenia in patients with liver cirrhosis. These findings suggest that *B. ovatus* may play a pivotal role in preserving skeletal-muscle health by modulating valine bioavailability. Additionally, the study observed significant enrichment of LPS biosynthesis pathways within the gut microbiome of sarcopenic patients. Coupled with previous reports that *B. ovatus* can suppress LPS-induced inflammation (Tan et al., 2018), it is hypothesized that this species exerts both metabolic and immunomodulatory functions during the development of sarcopenia (Aliwa et al., 2023). Collectively, these results systematically illuminate the critical role of the gut–muscle axis in cirrhosis-associated sarcopenia and provide a theoretical basis for future microbiota-targeted interventions.

Through functional-prediction analyses, Yamamoto et al. delineated the metabolic footprint of the gut microbiome in chronic liver disease patients with low skeletal muscle mass index (L-SMI): the L-SMI group exhibited elevated nitrogen-metabolism pathways, reduced abundance of genes involved in amino acid metabolism—including branched-chain amino acid biosynthesis—and increased expression of genes related to carbohydrate metabolism (Yamamoto et al., 2022). This profile suggests that these microbial communities preferentially utilize carbohydrates as an energy source, while their capacity for amino acid synthesis is compromised. Despite active nitrogen metabolism, the diminished representation of key amino acid biosynthetic genes indicates that nitrogen is likely channelled toward the production of non-amino acid metabolites. Notably, the study also observed a significant increase in the abundance of genes associated with LPS biosynthesis in the L-SMI group, a finding consistent with the results of Efremova et al. in sarcopenic patients (Efremova et al., 2024), suggesting that the LPS pathway may represent a shared underlying mechanism in the pathogenesis of muscle loss. At the compositional level, the L-SMI group displayed a higher relative abundance of *Bacteroidetes* (Gram-negative) and a lower abundance of *Firmicutes* (Gram-positive), an ecological shift that provides a microbiological basis for the observed LPS enrichment and may exacerbate muscle wasting by promoting inflammatory responses (Yamamoto et al., 2022).

Collectively, Yamamoto’s study uncovers a putative dual mechanism by which the gut microbiota impairs muscle health at the pre-sarcopenic or subclinical stage of low SMI: on the one hand, diminished BCAA synthetic capacity may constrain muscle-protein synthesis; on the other, increased LPS production may accelerate muscle breakdown via pro-inflammatory pathways (Yamamoto et al., 2022). These findings extend the link between microbial dysbiosis and compromised muscle integrity to an earlier stage of sarcopenia.

Multi-omic investigations have collectively uncovered the central role of the gut microbiota in chronic liver disease-associated sarcopenia and have begun to sketch the pathophysiological outline of the “gut–muscle axis.” Although conclusions regarding foundational issues such as microbial α-diversity remain divergent—exemplified by the discrepancies between Efremova and Yamamoto—this variability likely stems from heterogeneity in patient inclusion criteria (a comprehensive sarcopenia diagnosis versus a single L-SMI metric) and methodological approaches. Across studies, however, there is striking functional convergence: gut microbial communities in sarcopenia or low-SMI states consistently display a pro-inflammatory phenotype (e.g., enrichment of LPS biosynthetic genes), metabolic dysregulation (insufficient BCAA synthesis, carbohydrate-preferential metabolism, excessive secondary bile-acid production), and depletion of specific protective taxa such as *Akkermansia* and *Bacteroides ovatus*.

Current studies rely primarily on correlative metagenomic and metabolomic analyses. While these omics approaches have successfully mapped potential disease pathways in a systematic manner, they are subject to several notable limitations: first, functional predictions (e.g., PICRUSt) may not accurately reflect actual enzymatic activities; second, correlative findings are insufficient to establish definitive cause-and-effect relationships; and lastly, the dynamic fluctuations of microbial metabolites and their direct molecular mechanisms of action on the host remain poorly understood.

#### 3.2.3 Cross-population comparison and limitations of gut–muscle axis disruption patterns

In sarcopenia research, disease characteristics are typically defined by comparison with healthy or age-matched non-sarcopenic controls. The core disruption involves alterations in microbial composition, including reduced abundance of beneficial taxa (e.g., Bifidobacterium adolescentis, Faecalibacterium prausnitzii) and expansion of potentially harmful bacteria, leading to downstream metabolic consequences such as insufficient short-chain fatty acid (e.g., butyrate) production, decreased synthesis of NAD^+^ precursors (e.g., nicotinic acid), dysregulated bile-acid metabolism, and reduced levels of aromatic amino acid precursors (e.g., shikimate). These alterations are mechanistically linked to declines in muscle mass and function via signaling pathways including ERK/MAPK and SIRT1/PGC-1α.

In studies of CLD-associated sarcopenia, however, the reference frame shifts to cirrhotic patients who have not developed sarcopenia. Within this specific pathological context, disturbances of the gut–muscle axis carry a more pronounced “liver-disease signature.” Although some core taxonomic shifts—such as Akkermansia depletion—overlap with general sarcopenia, the overall dysbiosis pattern is more distinctive (e.g., reduction of Bacteroides ovatus, expansion of Bacteroides fragilis) and the metabolic derangements are both quantitatively greater and qualitatively unique. This is exemplified by a marked increase in the proportion of secondary bile acids (e.g., DCA, LCA) and by a more pronounced liver-driven systemic inflammatory milieu (e.g., widespread enrichment of LPS biosynthesis genes).

### 3.3 From multiple weak signals to multi-dimensional networks: integration model and future perspectives of the liver–gut–muscle axis

Previous studies have revealed a complex interplay between the muscle–liver axis and the gut–muscle axis in sarcopenia associated with chronic liver diseases. A systems-level analysis by Aliwa et al. provided pivotal modeling evidence for this interplay: using a signature constructed by least absolute shrinkage and selection operator (LASSO) regression followed by multivariable logistic regression, they showed that—although individual myokines such as irisin and myostatin were not significantly different in univariate analysis—their combined profile with specific bile-acid ratios and amino acid signatures constituted an independent multivariable feature associated with sarcopenia in cirrhosis (Efremova et al., 2024). This finding strongly indicates that the disorder’s core pathophysiology is not driven by a single dominant factor; rather, it arises from the synergistic accumulation of multiple low-amplitude signals across several biological axes.

How, then, are these “low-amplitude signals” generated and transmitted between organs? Their origin can be traced to the core pathological alterations of chronic liver disease. First, portal hypertension and hepatic dysfunction jointly compromise intestinal barrier integrity—including the gut vascular barrier—resulting in systemic translocation of microbial products such as lipopolysaccharide (LPS) and viable bacteria (Wiest et al., 2014; Spadoni et al., 2015; Sorribas et al., 2019; Albillos et al., 2020). Concurrently, disturbed hepatic bile-acid synthesis and dysbiotic gut microbiota interact to elevate the proportion of secondary bile acids such as deoxycholic acid (Lorenzo-Zúñiga et al., 2003; Kakiyama et al., 2013; Jiao et al., 2018; Albillos et al., 2020). Consequently, the gut microenvironment remodeled by CLD becomes a “factory” for aberrant signaling molecules.

Building on these observations, we propose an integrative “liver–gut–muscle axis” model (detailed mechanisms illustrated in **Supplementary Figure S1**). The central tenet of this model is that the aforementioned pathological changes do not conform to the classical “key-driver” paradigm; instead, they propagate as a cascade of low-amplitude signals along the liver-gut-muscle trajectory and are synergistically amplified. Ultimately, these signals interact with intrinsic, age-related metabolic defects in skeletal muscle (detailed in Chapter 2) and, through cumulative effects, overwhelm systemic metabolic homeostasis, driving muscle wasting. The establishment of this model signifies that the mechanistic framework must shift from seeking a single causal driver toward elucidating a multi-dimensional interactive network.

Building on the integrative “liver–gut–muscle axis” model, future investigations must move beyond association studies and achieve breakthroughs in three dimensions: causal inference, temporal dynamics, and personalized management. First, germ-free animal models and fecal microbiota transplantation should be employed to directly test the pathogenic or protective roles of defined taxa (e.g., *Bacteroides ovatus*) or their metabolites (e.g., secondary bile acids, valine), thereby establishing core causal relationships. Second, longitudinal cohorts with dense serial sampling and multi-omics time-series profiling are needed to dissect the causal networks and feedback loops linking microbiome–metabolome alterations to muscle loss. Ultimately, multi-omics and clinical data should be integrated through machine learning to construct disease endotypes and prognostic models, driving a shift from universal interventions toward personalized microbial therapies that target patient-specific pathogenic networks, thereby enabling early interception and precision management.

## 4 Omics-guided therapeutic strategies for sarcopenia: prospects and challenges

At present, the management of sarcopenia remains centered on exercise and nutritional support, and no targeted pharmacotherapy has been approved. Omics analyses demonstrate that diet constitutes one of the key determinants influencing the metabolome (Opazo et al., 2021). Exercise interventions—particularly resistance training—have been shown to improve muscle strength and physical performance (Cruz-Jentoft et al., 2014; Wu et al., 2021), presumably by activating the IGF-1/Akt–mTOR axis and enhancing protein synthesis (Song et al., 2017). Building on the aforementioned omics studies, this chapter critically reviews emerging therapeutic targets beyond conventional interventions and dissects their translational potential and inherent limitations.

### 4.1 Complexity of nutritional intervention: BCAA as a case study

Multiple studies have reported reduced plasma BCAA levels in sarcopenic individuals that correlate inversely with muscle mass, implying that BCAA supplementation—particularly leucine, the principal mTORC1 activator—might hold therapeutic potential (Lu et al., 2020; Hernández-Conde et al., 2021). Nevertheless, therapeutic efficacy has proved highly heterogeneous. Multi-omics work by Zuo et al. unveiled the underlying complexity: expression of BCAA-catabolizing enzymes (BCAT2 and BCKDHB) is down-regulated in sarcopenic muscle, leading to local accumulation of BCAAs and their keto-acids, which chronically overstimulate mTOR, provoke mitochondrial dysfunction, and accelerate muscle loss (Zuo et al., 2025). These findings indicate that in individuals with impaired BCAA catabolism, exogenous supplementation may fail to be incorporated into functional proteins and may even prove detrimental by exacerbating mTOR hyperactivation. Consistently, several clinical trials have shown that simply increasing dietary BCAA intake does not uniformly improve muscle function (Achison et al., 2022; Ebrahimi-Mousavi et al., 2022). Consequently, the benefit of BCAA intervention is critically dependent on an individual’s metabolic profile; future multi-center studies that incorporate metabolic stratification are required to identify responders and delineate potential risks.

### 4.2 Microbiome modulation: translating correlation into causation

Metabolomic and metagenomic surveys have consistently identified a depletion of butyrate-producing taxa—particularly *Bifidobacterium adolescentis* and *Faecalibacterium prausnitzii*—as a hallmark of sarcopenia (Guan et al., 2023; Zhang et al., 2025). Gnotobiotic and cell-based studies indicate that microbial-derived butyrate promotes myoblast proliferation via ERK1/2 activation (Guan et al., 2023), whereas *B. adolescentis* enhances mitochondrial function and suppresses proteolysis through a microbiota–niacin–NAD^+^ axis that engages the SIRT1/PGC-1α program (Zhang et al., 2025). Collectively, these data provide a compelling mechanistic rationale for microbiome-targeted interventions. Nevertheless, current evidence remains largely associative or derived from murine models; the causal relevance and precise molecular circuits in humans remain undefined. Whether next-generation consortia can engraft and maintain functional stability within the complex host ecosystem, and whether their efficacy is modulated by baseline microbiota configuration, diet, or co-medications, are critical questions that must be addressed before clinical translation.

### 4.3 New-target discovery: the long road from candidate molecule to licensed drug

Omics-driven studies have expanded the pool of druggable candidates for sarcopenia. For example, Lin et al. integrated multi-omics datasets to identify CD9 as a putative regulator of mitochondrial function in muscle wasting and performed in silico screening to shortlist dapoxetine and other potential ligands (Yin et al., 2025). Such work underscores the power of computational biology in target discovery. Nevertheless, the journey from target validation to market authorization remains a high-risk, decade-long process. The pathophysiological relevance of CD9 in human sarcopenia remains unproven, and the specificity, efficacy, and long-term safety of the shortlisted compounds must be systematically evaluated across multiple animal strains. Subsequent preclinical and clinical development phases are fraught with additional hurdles, including dose-finding, off-target effects, and demonstrating meaningful functional endpoints in heterogeneous patient populations.

.

### 4.4 Summary and outlook: therapeutic challenges and precision medicine prospects guided by omics

Multi-omics profiling has provided a systems-level framework for deciphering the mechanisms of sarcopenia and has simultaneously highlighted the central challenges impeding its therapeutic translation into precision medicine. The three intervention avenues dissected in this review all face conspicuous bottlenecks: BCAA supplementation is hampered by marked inter-individual heterogeneity in response; microbiome-based modulation still lacks definitive causal evidence and a reproducible translational pipeline; and the development of novel molecular targets is constrained by lengthy timelines and high attrition rates common to drug-discovery programs.

To overcome these bottlenecks, future research must shift the paradigm from “single-target” manipulation to “systems-level” intervention and adhere to the following translational roadmap. First, causality must be consolidated: gene-editing and organoid models should be leveraged to validate key targets (e.g., CD9, BCAA-catabolizing enzymes) and to establish robust biomarkers. The core step is precise stratification—integrating multi-omic signatures to build a molecular taxonomy grounded in BCAA metabolic status, microbial functional profiles, and related pathways. The ultimate objective is therapeutic innovation: mechanism-driven combination strategies such as “metabolic tuning plus nutritional synergy” or “defined probiotic consortia” should be developed for specific subgroups, and their clinical value confirmed through biomarker-enriched trial designs.

## 5 Limitations and translational challenges of omics-driven sarcopenia research

Although multi-omics technologies have provided an unprecedented systems-level view of the mechanisms underpinning sarcopenia, significant limitations in study design and data interpretation continue to constrain their clinical translatability. This chapter systematically dissects the core challenges of current research paradigms and proposes constructive approaches to bridge the gap between association and causation.

### 5.1 Diagnostic heterogeneity and technical barriers

The comparability and generalizability of multi-omics findings are first constrained by the intrinsic heterogeneity of sarcopenia definitions. Current consensus frameworks—such as those issued by AWGS and EWGSOP—apply divergent cut-offs for muscle mass, strength, and physical performance, resulting in inconsistent study populations and impeding data pooling and meta-analyses. At the technical level, both access and accuracy of diagnostic tools remain problematic. DXA is considered the gold standard for quantifying muscle mass, yet its high cost and operational complexity limit scalability in large cohorts or primary-care settings. Conversely, BIA is inexpensive and portable, but its readings are easily confounded by hydration status and other physiological variables, necessitating further improvements in reliability and validity.

### 5.2 Intrinsic limitations of study design and methodology

The current body of evidence carries a high risk of ecological fallacy, primarily rooted in study design:

#### 5.2.1 The challenge of causal inference

The vast majority of studies are cross-sectional, revealing only statistical associations between molecular markers and sarcopenia phenotypes without establishing temporal causality or direction (Lu et al., 2020). For example, it remains unclear whether observed gut dysbiosis is a driver of sarcopenia or a consequence of muscle loss and reduced physical activity (Efremova et al., 2024).

#### 5.2.2 Inadequate sample representativeness and statistical power

Many studies enroll limited samples (n < 100) and recruit specific ethnic or regional populations, introducing selection bias that markedly weakens external validity (Lu et al., 2020; Marques et al., 2023; Guo et al., 2024). Small sample sizes also reduce statistical power, making it difficult to detect true associations of modest effect size and increasing the likelihood of false-positive findings.

#### 5.2.3 Insufficient control of confounders

As an age-related disorder, sarcopenia is strongly influenced by diet, comorbidities, medications, and lifestyle factors. Notably, diet—the foremost environmental determinant of both the metabolome and the microbiome—introduces substantial confounding when not accurately recorded and controlled, obscuring the attribution of observed omics signals (Guo et al., 2024).

#### 5.2.4 Lack of standardization across technology platforms

Methodological limitations are inherent to omics technologies. In metabolomics, for instance, the absence of harmonized sample-preparation and separation workflows on LC-MS platforms precludes unbiased, comprehensive coverage of the structurally diverse plasma metabolome, producing marked disparities in metabolite panels across studies and compromising reproducibility and comparability (Opazo et al., 2021; Marques et al., 2023).

#### 5.2.5 Questionable clinical relevance of animal models

Some mechanistic studies rely on dexamethasone- or other drug-induced acute muscle atrophy models (Zhang et al., 2025). Although these models rapidly recapitulate accelerated protein degradation, they differ fundamentally from human age-related, slowly progressive sarcopenia, thereby limiting the translational value of their findings.

### 5.3 Complexity of interpretation and clinical translation

At the interpretation stage, challenges are equally formidable. First, sarcopenia frequently co-exists with obesity (sarcopenic obesity), and its distinct meta-inflammatory background can mask or confound the molecular signature of pure sarcopenia (Opazo et al., 2021). Second, most studies have not comprehensively examined how key biological variables—such as age and sex—modulate core molecular pathways, thereby overlooking the disorder’s intrinsic heterogeneity (Zhang et al., 2025). Collectively, these limitations prevent omics investigations from establishing reliable cause-and-effect relationships. Overcoming this fundamental bottleneck requires a paradigm shift from passive observation to active intervention and experimental validation.

### 5.4 Knowledge gap: the still-slender omics foundation of the field

It must first be stated that the molecular framework elaborated in this review rests on a small, currently available body of omics literature. Compared with other age-related disorders, high-quality multi-omics investigations devoted specifically to sarcopenia, especially when complicated by chronic liver disease, remain scarce and geographically scattered. This fundamental limitation has two immediate consequences. First, the core pathological pillars summarized here—such as proteostatic imbalance and mitochondrial dysfunction—are supported by existing data but likely represent only a subset of key nodes within the wider pathological network; many secondary pathways or subtype-specific mechanisms remain unknown. Second, the lack of sufficient independent cohorts for cross-validation and meta-analysis leaves the reliability of numerous “promising” omic signals in question. The mechanistic map presented should therefore be regarded as a dynamic and evolving scaffold whose completion is contingent upon the emergence of more systematic, large-scale omic studies.

### 5.5 Toward causal validation: an experimental roadmap from association to mechanism

At present, sarcopenia-omics overwhelmingly generates correlative hypotheses; the critical bottleneck to translation is establishing definitive biological mechanisms. Future investigations must therefore pivot from descriptive association to mechanistic validation. To this end, we propose a multi-tier functional validation framework:

#### 5.5.1 Gene-level causality

Use CRISPR/Cas9 to precisely manipulate candidate genes (e.g., CD9, BCAT2) in myoblasts or iPSC-derived myotubes and directly assess their necessity for mitochondrial function, protein turnover, and myogenic differentiation.

#### 5.5.2 Dynamic mapping of metabolic pathways

Apply stable-isotope labeling (e.g., ¹³C-leucine) together with metabolic-flux analysis to trace the fate and flow of key metabolites in muscle tissue, and couple these measurements to exogenous-intervention experiments that quantitatively evaluate physiological output.

#### 5.5.3 Physiological validation of systemic crosstalk

To dissect system-level mechanisms such as the gut–muscle axis, develop more predictive models—including patient-derived three-dimensional muscle organoids, gut-muscle-on-a-chip co-culture systems, and naturally ageing animal models—that allow complex multi-organ interactions to be tested in a physiologically relevant context.

### 5.6 Summary and action framework

In summary, omics research on sarcopenia stands at a critical crossroads where phenomenological description must give way to mechanistic insight. To overcome current bottlenecks, a systematic integration strategy is essential. First, priority should be given to launching large-scale, multinational, multicenter prospective cohort programs that address the fundamental problems of insufficient study numbers and fragmented data. To ensure the effectiveness of such initiatives, coordinated progress is required on three fronts: 1) standardization—achieving international consensus on diagnostic and omics workflows to enable data harmonization and comparison; 2) study design—rigorous control of confounders to strengthen causal inference; and 3) mechanistic dissection—mandating functional validation as an integral step in data interpretation to bridge the gap between association and causation. Only through this multi-level strategy can the current wealth of omics data be translated into reliable biological insights and actionable precision targets.

## 6 Conclusions and future directions

### 6.1 Core theoretical contributions

By constructing a “common–specific” dual-level framework, this review has systematically integrated multi-omic evidence and reconceptualized sarcopenia from a muscle-limited disorder to a systemic condition driven by multi-network dysregulation. The conceptual advance lies not only in delineating a “common” pathological backbone—comprising proteostatic imbalance, mitochondrial dysfunction, and other core cellular events—but also, by dissecting chronic liver disease as a comorbidity model, in uncovering disease-specific cross-organ axis (muscle–liver and gut–muscle) interactions that operate under particular pathological conditions. Grounded in these insights, our “synergistic accumulation of multiple low-amplitude signals” hypothesis challenges the traditional “single key-driver” paradigm and offers a new conceptual model for understanding the complex etiology of sarcopenia, especially when complicated by chronic disease.

### 6.2 Toward a next-generation paradigm: from association maps to mechanistic dissection

Sarcopenia research now stands at a pivotal inflection point where phenomenological description must yield to mechanistic understanding. Molecular network atlases built by omics technologies have provided an essential foundation for appreciating disease complexity, yet the overwhelming majority of findings remain correlational. Genuine breakthroughs will require paradigm innovation along three axes: 1) Research dimension: transition from static association to dynamic mechanism. Deploying single-cell multi-omics, spatial transcriptomics, and allied technologies should reveal the spatiotemporal dynamics of molecular networks within specific cellular subpopulations and tissue micro-environments, while CRISPR editing and isotopic flux analyses will be used to establish causal links. 2) Interventional strategy: move beyond the traditional “single-target” mindset toward network-centric, molecular-subtype-guided remodeling. Integrating multi-omic signatures to define disease endotypes will enable the design of combination regimens tailored to discrete patient subgroups, thereby achieving system-level correction of dysregulated biological networks. 3) Methodological framework: construct computational-biology models that fuse multi-omic datasets with longitudinal clinical information. Such models can simulate the dynamic trajectory of disease, forecast treatment-response paths, and furnish a theoretical scaffold for truly precision medicine.

This multi-dimensional paradigm shift points sarcometry research in a new direction: moving from constructing static molecular maps to building dynamic, predictive disease models, ultimately.

## Supplementary material

Supplementary Figure 1 Pathogenesis of Sarcopenia: From Fundamental Pathways to Liver Disease Comorbidity. On the right side of the figure, we present the potential molecular mechanisms of sarcopenia based on omics data without differentiating between the presence or absence of comorbidities; on the left side of the figure, we present the potential molecular mechanisms of sarcopenia based on omics data in the context of the existence of chronic liver disease comorbidity. CLD: Chronic liver disease. GDF-15: Growth Differentiation Factor 15; LPO: Lipid Peroxide; CHI3L1: Chitinase-3-like protein 1; *B. ovatus: Bacteroides ovatus; B. fragilis: Bacteroides fragilis; B. marseille: Blautia marseille; V. parvula: Veillonella parvula; B. adolescentis: Bifidobacterium adolescentis; P. succinatutens: Phascolarctobacterium succinatutens; P. faecium: Phascolarctobacterium faecium; B. longum: Bifidobacterium longum; B. pseudocatenulatum: Bifidobacterium pseudocatenulatum*; BCAAs: Branched-chain amino acids; mTOR mammalian target of rapamycin; ROS: Reactive oxygen species; VDR-DBD: Vitamin D receptor DNA-binding domain; NA: Nicotinic acid. LPS: Lipopolysaccharide; CA: Cholic acid; DCA: Deoxycholic acid; LCA: Lithocholic acid. (Created in BioRender. HUI, X. (2026) https://BioRender.com/nd3nydj.)

## Glossary

EWGSOP: European Working Group on Sarcopenia in Older People
FNIH: Foundation for the National Institutes of Health
IWGS: International Working Group on Sarcopenia
AWGS: Asian Working Group for Sarcopenia
GLIS: Global Leadership Initiative on Sarcopenia
CT: Computed tomography
MRI: Magnetic Resonance Imaging
SMA: skeletal muscle area
SMI: skeletal muscle index
MRA: Muscle radiation attenuation
DXA: Dual-energy X-ray absorptiometry
BIA: Bioelectrical impedance analysis
5-TCS: 5-time chair stand
SPPB: Short Physical Performance Battery
SARC-F: Strength, Assistance with walking, Rise from a chair, Climb stairs, and Falls
CLD: Chronic liver disease
GBD: Global Burden of Disease
ALD: alcohol-associated liver disease
MASLD: Metabolic dysfunction-associated steatotic liver disease
HCC: Hepatocellular carcinoma
LFI: Liver frailty index
ACLF: Acute-on-chronic liver failure
IGF-1: Insulin-like growth factor-1
MPS: Muscle protein synthesis
MPB: Muscle protein breakdown
DEGs: Differentially expressed genes
mTOR: Mammalian target of rapamycin
PKA: Protein Kinase A
EAAs: Essential amino acids
TA: Tibialis anterior
SOL: Soleus muscle
EDL: Extensor digitorum longus
BCAA: Branched-chain amino acid
BCKAs: Branched-chain α-keto acids
LC-FAs: Long-chain fatty acids
VLC-FAs: Very-long-chain fatty acids
ECI1: Enoyl-CoA isomerase 1
THIM: acetyl-CoA carboxylase carboxyl-transferase α-subunit
ECHD1: Ethylmalonyl-CoA decarboxylase
ATP: Adenosine Triphosphate
AMP: Adenosine Monophosphate
PI 38:4: Phosphatidylinositol 38:4
ETC: Mitochondrial electron transport chain
O₂•⁻: Superoxide anion
ROS: Reactive oxygen species
mPTP: Mitochondrial permeability transition pore
Cit: Citrulline
iNOS: Inducible nitric oxide synthase
NO: Nitric oxide
ONOO⁻: Oxidant peroxynitrite
SIRT1: Silent information regulator 1
PGC-1α: Peroxisome proliferator-activated receptor-γ
NRF1/2: Nuclear respiratory factors 1 and 2
TFAM: Mitochondrial transcription factor A
GSEA: Gene set enrichment analysis
PI3K-AKT: Phosphatidylinositol 3-Kinase –
AMPK: Adenosine Monophosphate-activated Protein Kinase
GWAS: Genome-wide association study
HLA: Human leukocyte antigen
NF-κB: Nuclear Factor kappa-light-chain-enhancer of activated B cells
HGS: Hand-grip strength
SCFA: Short-chain-fatty-acid
ASMI: appendicular skeletal muscle mass index
NMN: Nicotinamide mononucleotide
ILA: Indole-3-lactat
IAA: Indole-3-acetate
NA: Nicotinic acid
Try: Tyrosine
Ala: Alanine
Pro: Proline
Gly: Glycine
Asn: Asparagine
TCA: Taurocholic acid
TLCA: Ttaurolithocholic acid
CA: Cholic acid
CDCA: Cchenodeoxycholic acid
UDCA: Ursodeoxycholic acid
TCDCA: Taurochenodeoxycholic acid
TCA-3S: Taurocholic acid 3-sulfate
DCA: Deoxycholic acid
MPT: Mitochondrial permeability transition
TGR5: Takeda G protein-coupled receptor 5
IGFBP3: IGF-binding protein 3
MNA: Mini Nutritional Assessment
BMI: Body-mass index
HDL: High-density lipoprotein
25(OH)D₃: 25-hydroxyvitamin D₃
TUG: Timed Up-and-Go
DBD: DNA-binding domain
VDR: Vitamin D receptor
NAFLD: Non-alcoholic fatty liver disease
GSVA: Gene Set Variation Analysis
LDSC: LD-score regression
ALM: Appendicular lean mass
IMAT: intramuscular adipose tissue
PBMCs: Peripheral blood mononuclear cells cells
TNF-α: Tumor necrosis factor-alpha
TNF-R 1: Tumor necrosis factor receptor 1
sTNF-R1: Soluble TNF receptor 1
LPO: Lipid peroxidation
MAMC: Mid-arm muscle circumference
L-SMI: Low skeletal-muscle-mass index
N-SMI: Normal skeletal-muscle-mass index
LPS: Lipopolysaccharide
LCA: Lithocholic acid
T-UDCA: Tauroursodeoxycholic acid
BSH: Bile salt hydrolas
FXR: Farnesoid X Receptor
7α-HSDH: 7α-hydroxysteroid dehydrogenase
p70 S6K: p70 S6 kinase.
