# Supplementary material for: A review of omics studies in sarcopenia: from molecular mechanisms to hepatic-gut-muscle interactions in chronic liver disease comorbidity
Source: Front Cell Infect Microbiol. 2026 Jan 6;15:1710582. doi: 10.3389/fcimb.2025.1710582 (PMC12816193; doi:10.3389/fcimb.2025.1710582)
Supplement: Supplementary Figure 1 — Pathogenesis of Sarcopenia: From Fundamental Pathways to Liver Disease Comorbidity. On the right side of the figure, we present the potential molecular mechanisms of sarcopenia based on omics data without differentiating between the presence or absence of comorbidities; on the left side of the figure, we present the potential molecular mechanisms of sarcopenia based on omics data in the context of the existence of chronic liver disease comorbidity. CLD: Chronic liver disease. GDF-15: Growth Differentiation Factor 15; LPO: Lipid Peroxide; CHI3L1: Chitinase-3-like protein 1; B. ovatus: Bacteroides ovatus; B. fragilis: Bacteroides fragilis; B. marseille: Blautia marseille; V. parvula: Veillonella parvula; B. adolescentis: Bifidobacterium adolescentis; P. succinatutens: Phascolarctobacterium succinatutens; P. faecium: Phascolarctobacterium faecium; B. longum: Bifidobacterium longum; B. pseudocatenulatum: Bifidobacterium pseudocatenulatum; BCAAs: Branched-chain amino acids; mTOR mammalian target of rapamycin; ROS: Reactive oxygen species; VDR-DBD: Vitamin D receptor DNA-binding domain; NA: Nicotinic acid. LPS: Lipopolysaccharide; CA: Cholic acid; DCA: Deoxycholic acid; LCA: Lithocholic acid. (Created in BioRender. HUI, X. (2026) https://BioRender.com/nd3nydj.) [file DataSheet1.docx]

Supplementary Figures and Tables

## Supplementary Tables

Table S 1: Summary of Clinical and Multi-omics Study Characteristics in Sarcopenia Research.

| Author/  Year/  Country | Sample size | Experimental group  vs  control group | Specimen type | Diagnostic methods | Diagnostic criteria for sarcopenia | Multi-omics analysis  （Yes/No） | Types of omics analysis | Omics analysis platform |
| --- | --- | --- | --- | --- | --- | --- | --- | --- |
| Zhang/  2025/  China | 80 | Sar group（n=43）  vs  Con group (n=37) | Fecal, Blood | Grip strength,ASM,5TCS. | AWGS 2019 | Yes | Metagenomic Analysis | Illumina NovaSeq 6000 platform |
|  |  |  |  |  |  |  | Metabolomics Analysis | UPLC-MS |
| Yin/  2025/  China | ≤25^＊^(male) | model grou (SAM-P8) vs NC group (SAM-R1) | Skeletal muscle | Muscle endurance,The maximum grip strength. | N/A | Yes | Proteomic Analysis | N/A |
|  |  |  |  |  |  |  | Transcriptomics Analysis | N/A |
| Guo/  2024/  China | 15 | Low HGS (n=8)  vs HGS (n=7) | Fecal, Blood | HGS,ASM,5TCS,4-m gait speed,SPPB. | AWGS 2019 | Yes | Metabolomics Analysis | LC-MS |
|  |  |  |  |  |  |  | Microbiome Analysis | Illumina MiSeq System |
| Pergande/  2024/  United States | 12(male) | Old monkeys(n=4) vs middle-age monkeys(n=4) vs young monkeys(n=4) | Vastus lateralis skeleta | BMI | N/A | Yes | Proteomic Analysis | LC-MS/MS |
|  |  |  |  |  |  |  | Metabolomics Analysis | LC - Q - TOF MS/MS |
| Zuo/  2023/  China | 60 | S（n=20）  vs  PS（n=20） vs HA（n=20） | Vastus lateralis muscle | Grip strength,SMI,6-m gait speed. | AWGS 2019 | Yes | Transcriptome Analysis | Illumina sequencing platform |
|  |  |  |  |  |  |  | Proteomics Analysis | LC-MS/MS |
|  |  |  |  |  |  |  | Metabolomics Analysis | LC-MS/MS |
| Marques/  2023/  Germany | 22 | Sarcopenic (n=8)  Vs  Non-sarcopenic (n=14) | Plasma | HGS,ALM,SMI, z-score. | EWGSOP2 | No | Metabolomic  Analysis | HPLC-MS/MS |
| Guan/  2023/  China | 15 | With sarcopenia  (n = 7)  vs Without sarcopenia  (n = 8) | Fecal | Grip strength,ASM,ASMI,6 m gait speed. | AWGS 2019 | No | Transcriptomic Analysis | Illumina sequencing platform |
|  |  |  |  |  |  |  | Metabolomics Analysis | UPLC-MS/MS |
|  |  |  |  |  |  |  | Microbiome Analysis | Illumina MiSeq high throughput sequencing |
| He/  2023/  China | 63 | SAR（n = 32）  vs CON（n =31） | Fecal, Blood | Grip strength,ASM,5TCS,4-metre walk speed,SPPB. | AWGS 2019 | Yes | Shotgun Metagenomic Sequencing | Illumina HiSeq 2,500 device |
|  |  |  |  |  |  |  | Metabolomics Analysis | LC–MS |
| Opazo/  2021/  Chile | 41 | sarcopenic（n=20） vs non-sarcopenic  (n=21) | Blood | HGS,SMI,ASM. | EWGSOP1 | No | Metabolomics Analysis | UHPLC-ESI–MS/MS |
| Lu/  2020/  Singapore | 189 | Sarcopenia（n=87）  vs Non-sarcopenic (n=102) | Blood | Lower limb strength,6-m gait speed test. | AWGS2014 | No | Transcriptomics  Analysis | Illumina human HT-12 V4.0 bead chip plat-  form |
|  |  |  |  |  |  |  | Metabonomics Analysis | LC-MS/MS |

＊: The specific quantity varies depending on the experiment; Sar: Sarcopenia; Con: control; S:Sarcopenic; PS: Possibly sarcopenic; HA: healthy aged; ASM: AppendicularSkeletal Muscle Mass; 5TCS: 5-Time Chair Stand; HGS: Hand Grip Strength; SMI: Skeletal Muscle Index; ALM: Appendicular LeanMass; ASMI: Appendicular Skeletal Muscle Mass Index; BMI:Body Mass Index; SPPB: Short Physical Performance Battery; UPLC-MS: Ultra-Performance Liquid Chromatography–Mass Spectrometry; LC-MS: Liquid Chromatography–Mass Spectrometry; LC-MS/MS:Liquid Chromatography–Tandem Mass Spectrometry; HPLC-MS/MS:Targeted High-Performance Liquid Chromatography-Tandem Mass Spectrometry; UHPLC-ESI–MS/MS: Ultra-High-Performance Liquid Chromatography–Electrospray Ionization–Tandem Mass Spectrometry; LC–Q–TOF MS/MS: Liquid Chromatography Quadrupole Time-of-Flight Tandem Mass Spectrometry; N/A: “Not Applicable” or “Not Available”.

Table S 2: Cross-Omics Evidence Integration for Core Pathological Mechanisms in Sarcopenia.

| Core biological themes/ pathways | | Genomic evidence | Transcription/proteomics evidence | Metabolomics/microbiomics evidence | Non-genomic evidence supplementation | Integration findings |
| --- | --- | --- | --- | --- | --- | --- |
| Protein homeostasis imbalance | | N/A | 1.Transcription：  (1).The genes related to threonine/lysine metabolism were significantly downregulated;  (2).The analysis of blood samples revealed that the differentially expressed genes between patients with sarcopenia and those without were significantly enriched in the mTOR signaling pathway, the protein ubiquitination degradation pathway, and the PKA signal cascade reaction;  (3).The analysis of the blood sample revealed that the mTOR signaling pathway was significantly suppressed. | Metabolomics:  (1).The plasma concentrations of the seven essential amino acids (methionine, lysine, phenylalanine, threonine, and the branched-chain amino acids leucine, isoleucine, and valine) were significantly reduced;  (2).The levels of plasma glutamine and methionine were significantly increased, while other protein and non-proteinogenic amino acids such as leucine and glutamic acid were significantly decreased. | PCR-Rodents: The expressions of FoxO3, Atrogin-1 and MuRF-1 in the TA, SOL and EDL of aged mice were significantly increased. | Abnormalities in amino acid metabolism can promote the development of sarcopenia by disrupting the balance between muscle protein synthesis and breakdown. |
|  |  |  | 2.Proteomics-Primates: Actin, myosin, integrin and collagen levels have significantly decreased. |  |  |  |
| Mitochondrial dysfunction and quality control impairment | 1.BCAA catabolism defect | Genetic variations (SNPs) that affect muscle mass are enriched in the BCAA metabolic pathway. | 1.Transcription:  (2).The mRNA expressions of key enzymes involved in BCAA catabolism, such as BCAT2 and BCKDHB, were significantly decreased;  (3).The genes related to mitochondrial oxidative phosphorylation, the TCA cycle, and fatty acid β-oxidation were widely downregulated | Metabolomics:  1.Significant accumulation of BCAAs and their (BCKAs was observed in the skeletal muscle tissue;  2.The levels of high-energy phosphate compounds (ATP, ADP, phosphocreatine) have significantly decreased. | N/A | Defective BCAA catabolism sustains mTOR activation, triggering a mitochondrial cascade and insulin resistance, which collectively drive progressive skeletal muscle decline in sarcopenia. |
|  |  |  | 2.Proteomics:  (1).The protein abundances of BCAT2 and BCKDHB decreased simultaneously;  (2).Decrease in the protein abundance of subunits I-V of the electron transport chain complex. |  |  |  |
|  |  |  | 3.Transcription/proteomics:  (1).The phosphorylation levels of mTOR and its downstream effectors p70S6K and S6 were significantly increased;  (2).The phosphorylation level of the key autophagy initiation protein ULK1 at the Ser757 site increased, while the ratio of autophagy marker LC3-II/LC3-I decreased, and p62 accumulated.  (3).The phosphorylation level of IRS1 (Ser307) increased, while the phosphorylation level of Akt (Ser473) decreased. |  |  |  |
|  | 2.Mitochondrial fatty acid oxidation dysfunction leads to energy metabolism disorders. | N/A | 1.Transcription:  The mRNA expression levels of key enzymes in fatty acid β-oxidation, such as CPT1B and HADHB, were significantly downregulated in the skeletal muscles of patients with sarcopenia. | 1.Metabolomics：  (1).The extremely VLC-FA and their ω-oxidation products, dihydroxy carnitine (Carn. DC), were significantly increased;  (2).The baseline levels of the ω-6 series basic LCFA have significantly increased;  (3).The expression of enzymes related to fatty acid oxidation (such as CPT1B, HADHB) is downregulated, and intermediate fatty acid products (such as caproic acid, octanoic acid) accumulate;  (4).Quantitative enrichment analysis revealed that the metabolic profile of sarcopenia was highly consistent with that of various genetic mitochondrial fatty acid oxidation disorders (such as CPT II deficiency, LCHAD, VLCAD). | N/A | Mitochondrial fatty acid oxidation dysfunction may be one of the conserved pathogenic mechanisms of sarcopenia across species. |
|  |  |  | 2.Proteomics-Primates:  (1).Significant changes occurred in the expression of multiple key enzymes in the fatty acid β-oxidation pathway;  (2).The expression of enzymes related to ATP production (such as the electron transport chain complexes) shows compensatory increase, while AMP metabolic enzymes are downregulated. | 2.Metabolomics-Primates:  (1).In vastus lateralis muscle, palmitoyl carnitine and arachidonic acid carnitine markedly accumulated, with concomitant alterations in acetyl carnitine—the key β-oxidation product;  (2).Key energy metabolites—including carnitine, trimethyllysine, phosphocreatine, and phosphatidylinositol 38:4 (PI 38:4)—were significantly reduced in middle-aged and elderly animals. |  |  |
|  | 3.The ROS-ONOO⁻ axis attacks mtDNA, leading to mitochondrial damage. | N/A | N/A | Metabolomics:  The plasma Cit level in patients with sarcopenia is significantly elevated, and citrulline is the most critical variable for distinguishing the metabolic characteristics of sarcopenia patients. The metabolic pathway to which citrulline belongs is significantly enriched in sarcopenia. | Cit is a byproduct of iNOS-catalyzed NO synthesis from L-arginine. Excess NO reacts with ETC-derived O₂•⁻ to form peroxynitrite (ONOO⁻), which irreversibly inhibits complex I and disrupts mitochondrial function, aggravating cellular energy crisis. | Elevated Cit reflects heightened iNOS activity and NO overproduction. NO combines with mitochondrial superoxide to form cytotoxic ONOO⁻, exacerbating oxidative stress, impairing mitochondrial function, and ultimately driving muscle atrophy. |
|  | 4.Abnormal regulation of CD9 impairs mitochondrial biosynthesis | N/A | Transcription/proteomics;  (1).*CD9* is a key gene closely related to sarcopenia;  (2).CD 9 regulates aerobic respiration and ATP biosynthesis, and is involved in mitochondrial biosynthesis and oxidative phosphorylation. | N/A | Immunofluorescence staining/qPCR:  The expression level of CD9 in skeletal muscle significantly decreases during the aging process. | CD9 potentially contributes to sarcopenia pathogenesis by modulating mitochondrial biogenesis and oxidative phosphorylation. |
| Inflammatory aging | | N/A | Transcription:  (1).In patients with sarcopenia, the genes *F0LR3, NAMPT, CXCR4*, and *VNN3* were significantly upregulated;  (2).The NF-κB signaling pathway and the B-cell receptor signaling pathway were significantly inhibited, while the PI3K/AKT signaling pathway was significantly activated；  (3).The expression of nicotine response-related genes (*ABAT* and *KCNK1*) in patients with muscle atrophy was downregulated. | Metabolomics：  (1).In patients with sarcopenia, the plasma levels of various essential amino acids - lysine, methionine, phenylalanine, threonine, as well as all branched-chain amino acids - leucine, isoleucine and valine - were significantly reduced;  (2).Functional vitamin B6 deficiency and decreased choline levels;  (3).The levels of nicotine metabolites related to smoking have increased. | N/A | Chronic low-grade inflammatory state promotes the progressive loss of muscle mass and function through multiple mechanisms such as activating immune signals, reshaping metabolic homeostasis, and enhancing protein breakdown. |
| Gut-muscle axis | 1.Lack of SCFAs inhibits the proliferation of muscle cells. | N/A | Transcription:  Genes related to the PI3K-Akt pathway were significantly enriched. | Metabolomics:  (1).The decrease in bifidobacteria, particularly those such as *B. longum, B. pseudocatenulatum* and *B. adolescentis*, which are capable of producing SCFAs;  (2).The abundance of the butyrate-producing bacteria *Faecalibacterium prausnitzii* and *Eubacteriaceae* decreased significantly;  (3).The content of butyric acid in the feces has significantly decreased. | PCR/Western Blot:  After butyrate treatment, Myf5 and MyoD were upregulated at both mRNA and protein levels in C2C12 cells. | Butyrate, influenced by gut microbiota, promotes C2C12 myoblast proliferation via ERK/MAPK activation and Myf5/MyoD upregulation, thereby enhancing skeletal muscle regeneration. |
|  | 2.Disorder of the gut microbiota - niacin - NAD+ axis impairs muscle regeneration and mitochondrial homeostasis. | Genomics:  In patients with sarcopenia, the activities of five key pathways involved in NAD+ metabolism were significantly reduced. | N/A | 1.Metabolomics:  The content of NA in the serum of patients with sarcopenia was significantly decreased. | PCR-Rodents：  1.The expressions of FoxO3, Atrogin-1 and MuRF-1 in the TA, SOL and EDL of aged mice were significantly increased;  2.The SIRT1, PGC-1a, Tfam, NRF1, NRF2 genes and mtDNA genes in the TA and SOL of aged mice were significantly decreased. | The NA produced by *B. adolescents* can comprehensively improve the function of aging skeletal muscles by increasing the level of NAD+. |
|  |  |  |  | 2.Microbiomics:  *B. adolescentis* reduction. | 2.Others:  (1).*B. adolescence*, NA is produced by NA phosphoribosyltransferase (k00763);  (2).Supplementing with *B.adolescentis* or directly supplementing with NA can significantly increase the number of PAX7⁺ MuSCs and the proportion of myotrophic protein-positive muscle fibers, and significantly elevate the tissue NAD⁺ level. |  |
|  | 3.The dysfunction of the gut microbiota-bile acid axis in mitochondrial toxicity and signal interference. | N/A | N/A | Metabolomics:  The levels of bile acids (such as TCA, TLCA) positively correlated with skeletal muscle index and grip strength decreased, while the levels of bile acids (such as CA, CDCA, UDCA) negatively correlated with muscle loss increased; | (1).High bile acid concentrations induce MPT.  (2).Gut dysbiosis (e.g., reduced BSH activity) disrupts bile acid homeostasis, leading to FXR antagonism (e.g., TβMCA accumulation). This inhibits intestinal FXR-FGF15 signaling, suppressing muscle anabolic pathways while upregulating atrophy genes, ultimately driving sarcopenia. | Bile acids participate in the occurrence and development of sarcopenia through multiple mechanisms. |
|  | 4.Abnormalities in carbohydrate metabolism enzymes lead to insufficient energy supply. | Genomics:  (1).The amino acid metabolic pathways of the intestinal flora in patients with sarcopenia have undergone significant changes;  (2). the expression of functional genes related to carbohydrate-active enzymes (CAZyme) is abnormal. | N/A | Metabolomics analysis:  The level of shikimic acid in the feces of patients with sarcopenia was significantly decreased. | *Phascolarctobacterium faecium* can promote the production of phydroxybenzoic acid, thereby influencing the biosynthesis of phenylalanine, tyrosine and tryptophan. | Gut microbiota dysbiosis, characterized by reduced beneficial bacteria and increased harmful bacteria, impairs carbohydrate metabolism and depletes beneficial metabolites (e.g., shikimic acid), thereby disrupting energy homeostasis and promoting sarcopenia. |
| Systemic synergistic factors: Hormones and nutritional metabolism | 1.The decline in IGF-I levels weakens the mitochondrial protection mechanism. | N/A | N/A | Metabolomics:  (1).IGF-I exerts a significant indirect effect on sarcopenia by influencing VLC-FA；  (2).IGF-I is negatively correlated with three types of VLC-FA, namely NEFA 26:2, NEFA 24:4 and NEFA 24:2, as well as Cit. Moreover, the ratio of IGF-I to IGFBP3 is also negatively correlated with various long-chain and very long-chain non-esterified fatty acids. | Low levels of IGF-I are associated with mitochondrial dysfunction in aged rats. | IGF-I indirectly participates in the occurrence of sarcopenia. |
|  | 2.Nutritional and metabolic disorders exacerbate the progression of sarcopenia through multiple mechanisms. | N/A | Transcriptomics:  The PIM1 gene was significantly downregulated in patients with sarcopenia | Metabolomics:  (1).BMI and leptin are negatively correlated with sarcopenia, while adiponectin and HDL are positively correlated with sarcopenia;  (2).Muscle atrophy is significantly negatively correlated with multiple levels of essential amino acids, including lysine, methionine, phenylalanine, threonine, BCAA and choline. | (1).The MNA score and the risk of malnutrition are independently associated with the presence of sarcopenia;  (2).The PIM1 kinase can regulate downstream signaling pathways by directly interacting with the DNA binding domain of the vitamin D receptor. | It is suggested that nutrition plays a crucial role in maintaining muscle health, and vitamin D may promote the occurrence of sarcopenia by maintaining the integrity of signaling pathways. |

BCAA: Branched-chain amino acid; BCKA: Branched-chain keto acid; BCAT2: Branched-chain amino acid transaminase 2; BCKDHB: Branched-chain keto acid dehydrogenase E1 subunit beta; mTOR: Mechanistic target of rapamycin ; PKA: Protein kinase A; FoxO3: Forkhead box O3; MuRF-1: Muscle RING-finger protein-1; ULK1: Unc-51 like autophagy activating kinase 1 ; IRS1: Insulin receptor substrate 1; Akt: Protein kinase B; CPT1B: Carnitine palmitoyltransferase 1B; HADHB: Hydroxyacyl-CoA dehydrogenase trifunctional multienzyme complex subunit beta; VLC-FA: Very long-chain fatty acid ; Carn.DC: Dicarboxylic acylcarnitine; LCHAD: Long-chain 3-hydroxyacyl-CoA dehydrogenase; VLCAD: Very long-chain acyl-CoA dehydrogenase; ROS: Reactive oxygen species; ONOO⁻: Peroxynitrite; mtDNA: Mitochondrial DNA; iNOS: Inducible nitric oxide synthase ; NO: Nitric oxide; ETC: Electron transport chain; SCFA: Short-chain fatty acid; NAD⁺: Nicotinamide adenine dinucleotide; NA: Nicotinic acid; FXR: Farnesoid X receptor; TβMCA: Tauro-β-muricholic acid; FGF15: Fibroblast growth factor 15; CAZyme: Carbohydrate-active enzyme; IGF-I: Insulin-like growth factor I; IGFBP3: Insulin-like growth factor binding protein 3 ; PIM1: Pim-1 oncogene; TA: Tibialis anterior; SOL: Soleus; EDL: Extensor digitorum longus; MuSCs: Muscle stem cells; B. longum: Bifidobacterium longum; B. pseudocatenulatum: Bifidobacterium pseudocatenulatum; B. adolescentis: Bifidobacterium adolescentis; TCA: Taurocholic acid; TLCA: Taurolithocholic acid ; CA: Cholic acid; CDCA: Chenodeoxycholic acid; UDCA: Ursodeoxycholic acid; BSH: Bile salt hydrolase; MPT: Mitochondrial permeability transition; MNA: Mini Nutritional Assessment ; BMI: Body mass index; HDL: High-density lipoprotein; PCR: Polymerase chain reaction; qPCR: Quantitative real-time PCR. N/A: “Not Applicable” or “Not Available”.

Table S 3: Summary of Clinical and Multi-omics Study Characteristics in Sarcopenia Associated with Chronic Liver Disease.

| Author/  Year/  Country | Sample size | Specimen type | Diagnostic criteria for sarcopenia | Diagnostic criteria for sarcopenia | Types of omics analysis | Omics analysis platform |
| --- | --- | --- | --- | --- | --- | --- |
| Efremova/  2024/  Russia | 40 | Fecal | SMI | SMI :  Males<50 cm2/m2 ;  Fmales< 39 cm2/m2 | Microbiome Analysis | Illumina MiSeq |
| Nicholson/  2024/  UK | 56 | Blood, Vastus lateralis | N/A | N/A | Epigenomics Analysis | Infinium EPIC 850k Methylation array |
|  |  |  |  |  | Transcriptomics Analysis | Illumina NextSeq 500 |
|  |  |  |  |  | Immunophenotyping | MacsQuant X |
| Aliwa/  2023/  Austria | 175 | Blood, Fecal, Urine | SMI,HGS,Gait speed. | EWGSOP（2010） | Microbiomics Analysis | Illumina MiSeq |
|  |  |  |  |  | Metabolomics Analysis | UPLC-MS/MS |
|  |  |  |  |  |  | NMR |
| Xu/  2023/  China | 170 | N/A | N/A | N/A | Transcriptomics Analysis | Illumina high throughput sequencing platform |
| Yamamoto/  2022/  Japan | 69 | Fecal | SMI | SMI:  Males＜42 cm2/m2 Fmales＜38 cm2/m2 | Microbiome Analysis | Illumina MiSeq |
| Lu/  2022/  China | 136 (Male) | Plasma, Tumor tissue | SMI | SMI:  Males＜43.75 cm2/m2 | Metabolomics Analysis | LC-MS |
|  |  |  |  |  | Transcriptomics Analysis | Illumina |

HGS: Handgrip Strength; SMI: Skeletal Muscle Index; EWGSOP: European Working Group on Sarcopenia in Older People; UPLC‑MS/MS: Ultra-Performance Liquid Chromatography–Tandem Mass Spectrometry; NMR: Nuclear Magnetic Resonance; LC‑MS: Liquid Chromatography–Mass Spectrometry; N/A: “Not Applicable” or “Not Available”.

Table S 4: Cross-Omics Evidence Integration for Core Pathological Mechanisms in Sarcopenia Associated with Chronic Liver Disease.

| Core biological themes/ pathways | | Genomic evidence | Transcriptome/proteomics evidence | Metabolomics/microbiomics evidence | Non-genomic evidence | Integration findings |
| --- | --- | --- | --- | --- | --- | --- |
| The omics analysis and pathological remodeling of the muscle-liver axis. | 1.Metabolic-epigenetic co-regulation: Systemic disorder of the muscle-liver axis. | Genomics:  NAFLD shows a significant positive genetic correlation with grip strength and ALM. | Transcriptomics:  (1).Muscular dystrophy and NAFLD share significant overlaps in multiple key metabolic pathways, including lipid metabolism (such as fatty acid β-oxidation and cholesterol metabolism), oxidative stress (including ROS pathways and antioxidant stress responses), and energy metabolism (such as the TCA cycle and mitochondrial function);  (2).The metabolic pathways such as purine nucleotide salvage and lipoxygenase are significantly correlated in both diseases; the common genes of the two diseases mainly participate in biological processes such as ribonucleoprotein complex biosynthesis, ribosome biosynthesis, ncRNA processing, histone modification, and rRNA metabolism;  (3).NAFLD and sarcopenia show a significant positive correlation in their gene expression patterns；  (4).Four pairs of key regulatory genes with co-expression characteristics were identified: *HIF1A, ATG5, ADM* and *CST3* showed high expression in both diseases, while *BMP2, BMPR2, TFDP1* and *E2F6* showed low expression;  (5).The pleiotropic genes related to NAFLD and ALM/grip strength are significantly enriched in the liver, skeletal muscle, pancreas, blood and brain tissues, and are enriched in antigen presentation, cytokine signaling and mitochondrial function pathways. | N/A | N/A | The co-morbidity mechanism of sarcopenia and NAFLD involves a complex regulatory network composed of metabolic disorders, dysregulation of gene expression, and epigenetic modifications. |
|  | 2.The CHI3L1-LPO axis: The myogenic signal driving the malignant cycle of liver diseases. | N/A | 1.Transcriptome:  High plasma CHI3L1 levels were significantly positively correlated with abnormal liver PPAR signaling pathway, peroxisome function, and arachidonic acid metabolism. | Metabolomics:  Identifies 83 key metabolites, revealing that the increase in CHI3L1/sTNF-R1 is closely associated with lipid metabolism disorders such as sterol lipids and fatty acids. | N/A | In patients with sarcopenia, CHI3L1 is upregulated and secreted by skeletal muscle via the TNF-α/TNF‑R1 signaling pathway, protecting myocytes from damage, while concurrently promoting HCC progression by inducing the accumulation of LPO products. |
|  |  |  | 2.Proteomics:  In the patient cohort, the CHI3L1 protein derived from skeletal muscle and its soluble receptor sTNF-R1 were identified as key molecules, and their protein levels were significantly elevated. |  |  |  |
|  | 3.Systemic aging: The distal effects driven by the muscle-liver axis in chronic liver diseases. | Epigenomics:  The epigenetic age of skeletal muscles in CLD patients is significantly higher than that of the healthy control group. | Transcriptomics:  The expression of aging-related genes (SemMayo gene set) in the skeletal muscle tissues of CLD patients was significantly enriched. | N/A | (1).ELISA: The level of the aging marker GDF-15 significantly increased;  (2).Flow cytometry: Changes in immune aging of T cell and B cell subsets;  (3).IMAT measurement: Epigenetic abnormalities were significantly positively correlated with IMAT;  (4).The immune aging score (IMM-AGE score) of CLD patients reached twice that of the healthy control group. | Liver lesions, through epigenetic reprogramming, chronic inflammatory microenvironment and metabolic disorders, ultimately lead to muscle atrophy and immune dysfunction via the regulatory network of the muscle-liver axis. |
| The unique disordered and integrated model of the gut-muscle axis. | | Genomics:  (1).The sarcopenic cirrhosis gut microbiome displays pathway enrichment in LPS biosynthesis, carbohydrate digestion/absorption, folate synthesis, and citrate cycle, alongside depletion in antibiotic biosynthesis, biofilm formation, and pentose phosphate pathway;  (2).Bile acid metabolism associated gene abundance did not differ significantly between sarcopenic and non-sarcopenic groups;  (3) Nitrogen metabolism pathways were enriched in the SMI group;  (4) LPS biosynthesis genes were significantly upregulated in the L-SMI group. | Transcriptomics:  In the L-SMI group, the expression levels of genes related to amino acid metabolism were relatively low, while those related to carbohydrate metabolism were relatively high. | 1.Microbiomics:  (1). In patients with liver cirrhosis and sarcopenia, the relative abundances of bacterial groups such as *Bacteroides fragilis*, *Blautia marseille, Sutterella spp., and Veillonella parvula* significantly increased, while *Bacteroides ovatus* was more enriched in patients without sarcopenia;  (2).The relative abundances of *Proteobacteria* and *Bacteroides* were higher in the SMI group, while the relative abundance of *Firmicutes* and the ratio of Firmicutes to Bacteroidetes were lower;  (3).The relative abundances of *Coprobacillus, Catenibacterium* and *Clostridium* were lower in the SMI group, while the relative abundance of *Bacteroides* was higher. | (1).Akkermansia may maintain muscle mass by strengthening the intestinal barrier function, inhibiting LPS translocation and inflammatory responses；  (2).*Eggerthella*, by activating the Th17 inflammatory pathway and promoting secondary bile acid metabolism, etc., further exacerbates muscle catabolism；  (3).Elevated bile acids in chronic liver disease activate muscle TGR5, inducing oxidative stress and upregulating muscle-specific E3 ligases to activate ubiquitin-proteasome and autophagy pathways, culminating in muscle atrophy and loss;  (4).*B. ovatus* can inhibit the inflammatory response induced by lipopolysaccharide. | In the context of sarcopenia or L-SMI (low skeletal muscle mass) conditions, the gut microbiota generally exhibits pro-inflammatory phenotypes (such as enrichment of LPS biosynthesis genes), metabolic disorders (such as insufficient synthesis of BCAAs, preference for carbohydrate utilization, excessive production of secondary bile acids), and depletion of specific protective bacterial genera (such as *Akkermansia*, *Bacteroides ovatus*). |
|  |  |  |  | 2.Metabolomics:  (1).Patients with liver cirrhosis and sarcopenia showed significant increases in serum secondary bile acids and total LCA levels in feces, and several ratios reflecting the conversion from primary to secondary bile acids also significantly increased；  (2).A significant decrease in serum valine levels |  |  |

NAFLD: Non-alcoholic fatty liver disease; ALM: Appendicular lean mass; CLD: Chronic liver disease; L-SMI: Low skeletal muscle index; SMI: Skeletal muscle index; CHI3L1: Chitinase-3-like protein 1; LPO: Lipid peroxidation ; sTNF-R1: Soluble tumor necrosis factor receptor 1 ; HIF1A: Hypoxia inducible factor 1 subunit alpha; ATG5: Autophagy related 5; ADM: Adrenomedullin ; CST3: Cystatin C; BMP2: Bone morphogenetic protein 2 ; BMPR2: Bone morphogenetic protein receptor type 2; TFDP1: Transcription factor Dp-1; E2F6: E2F transcription factor 6; PPAR: Peroxisome proliferator-activated receptor; GDF-15: Growth differentiation factor 15; LPS: Lipopolysaccharide; BCAAs: Branched-chain amino acids ; TGR5: Takeda G protein-coupled receptor 5; Th17: T helper cell 17; IMAT: Intermuscular adipose tissue ; *B. ovatus: Bacteroides ovatus*; ELISA: Enzyme-linked immunosorbent assay; IMM-AGE score: Immune aging score. N/A: “Not Applicable” or “Not Available ”.

## Supplementary Figure


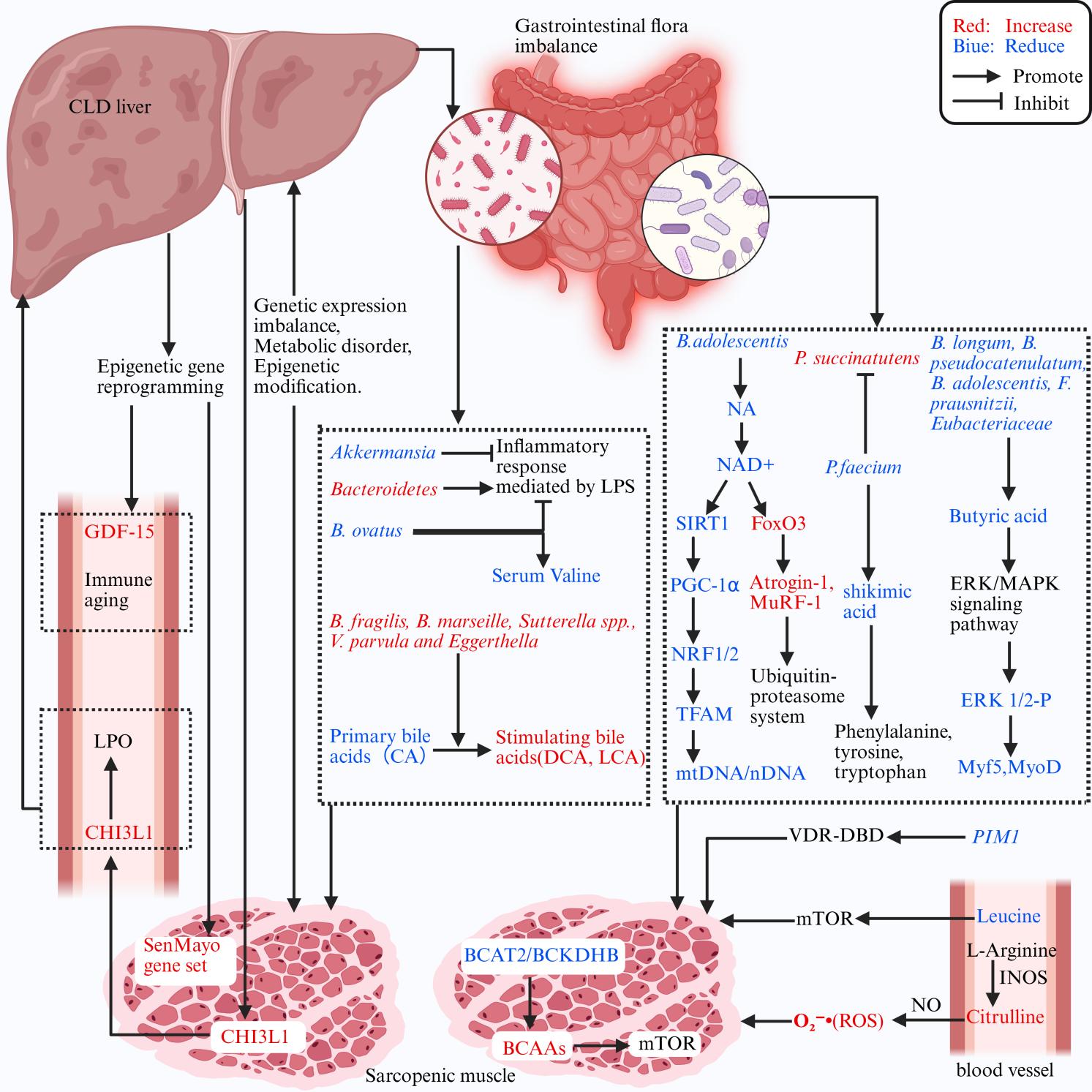


Figure S1: Pathogenesis of Sarcopenia: From Fundamental Pathways to Liver Disease Comorbidity. On the right side of the figure, we present the potential molecular mechanisms of sarcopenia based on omics data without differentiating between the presence or absence of comorbidities; on the left side of the figure, we present the potential molecular mechanisms of sarcopenia based on omics data in the context of the existence of chronic liver disease comorbidity. CLD: Chronic liver disease. GDF-15: Growth Differentiation Factor 15; LPO: Lipid Peroxide; CHI3L1: Chitinase-3-like protein 1; *B. ovatus: Bacteroides ovatus; B. fragilis: Bacteroides fragilis; B. marseille: Blautia marseille; V. parvula: Veillonella parvula; B. adolescentis: Bifidobacterium adolescentis; P. succinatutens: Phascolarctobacterium succinatutens; P. faecium: Phascolarctobacterium faecium; B. longum: Bifidobacterium longum; B. pseudocatenulatum: Bifidobacterium pseudocatenulatum*; BCAAs:Branched-chain amino acids; mTOR mammalian target of rapamycin; ROS: Reactive oxygen species; VDR-DBD: Vitamin D receptor DNA-binding domain; NA: Nicotinic acid. LPS: Lipopolysaccharide; CA: Cholic acid; DCA: Deoxycholic acid; LCA: Lithocholic acid. (Created in BioRender. HUI, X. (2026) https://BioRender.com/nd3nydj. )
